# Integrated genome mining and phytohormone profiling of six plant growth-promoting elite bacterial strains

**DOI:** 10.1007/s00203-025-04712-6

**Published:** 2026-01-21

**Authors:** Tairine Graziella Ercole, Rafaella Liviero, Leonardo Araujo Terra, Guilherme Julião Zocolo, Milena Serenato Klepa, Renan Augusto Ribeiro, Marco Antonio Nogueira, Mariangela Hungria

**Affiliations:** 1 Soil Biotechnology Laboratory, Embrapa Soja, C.P. 4006, Londrina, PR CEP 86.085-981 Brazil; 2 CNPq, SHIS, Conjunto B, Edifício Santos Dumont, Lago Sul, Federal District, Brasília, CEP 71.605-170 Brazil; 3 Department of Biochemistry and Biotechnology, Universidade Estadual de Londrina, C.P. 10011, Londrina, Paraná 86.057-970 Brazil; 4 Chemical Ecology Laboratory, Embrapa Soja, C.P. 4006, Londrina, PR CEP 86.085-981 Brazil; 5 Araucária Foundation, Comendador Franco Ave., C.P. 1341, Curitiba, PR CEP 80.215-090 Brazil

**Keywords:** PGPB (plant growth-promoting bacteria), Genome analysis, Targeted metabolomic, Phytohormones production, Taxonomic, Multifunctional bio-inputs

## Abstract

Plant growth-promoting bacteria may act by enhancing soil fertility, nutrient cycling, and pathogen suppression. We analyzed the genomes and metabolomes of six strains, *Chromobacterium violaceum* CNPSo 1954, *Pantoea agglomerans* CNPSo 2602, *Bacillus velezensis* CNPSo 2657, *Bacillus altitudinis* CNPSo 2658, *Bacillus safensis* CNPSo 2725, and the novel species *Pseudomonas* sp. CNPSo 2799. Genomic bioprospection revealed diverse biosynthetic gene clusters (BGCs) involved in secondary metabolites production, accounting for 4.26% of the total genome in strain CNPSo 2602 and 18.03% in strain CNPSo 2657. An average of 79 carbohydrate-active enzymes (CAZymes) were identified per genome, with glycoside hydrolases and glycosyltransferases accounting for more than 50% of all identified enzymes. The strains exhibited distinct antibiotic resistance profiles, ranging from three (CNPSo 2658 and CNPSo 2725) to 12 (CNPSo 2602). All strains carried the genes for tryptophan-biosynthesis, and targeted metabolomic analysis confirmed the production of the phytohormones indole-3-acetic (IAA), indole-3-butyric (IBA), indole-3-pyruvic acids (IPA), and L-tryptophan (TRP), with strain-specific variation in metabolic profiles. These strains exhibited multiple growth-promoting and biocontrol traits, highlighting a potential as multifunctional next-generation bio-inputs for sustainable agricultural applications.

**Graphical abstract:**

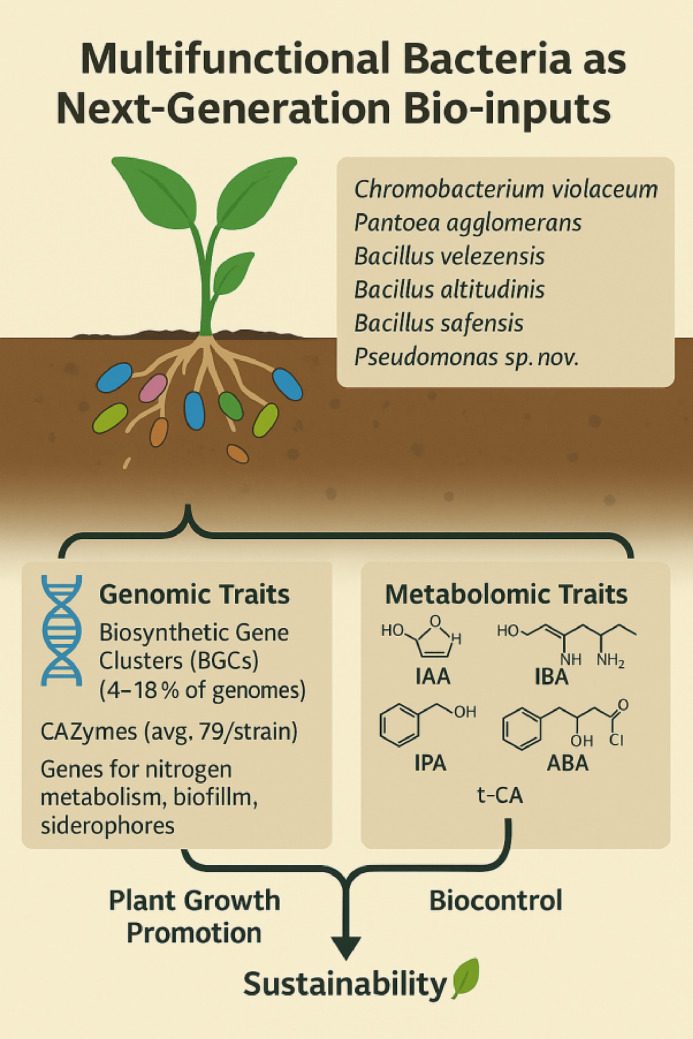

**Supplementary Information:**

The online version contains supplementary material available at 10.1007/s00203-025-04712-6.

## Introduction

Soil microorganisms play a key role in maintaining soil fertility by driving processes such as nutrient cycling, organic matter decomposition, and ecosystem balance (Fanai et al. 2024). They also influence soil structure, and the composition of microbial communities can shift substantially under different management practices (Zhao et al. 2025a). Among these microorganisms, plant growth-promoting bacteria (PGPB) represents a critical component of the soil microbiome. These bacteria have established a continuous, dynamic, and co-evolutionary relationship with plants, supporting their growth by facilitating the acquisition of essential nutrients and other beneficial compounds. In addition, certain bacterial species can suppress phytopathogens by releasing bioactive molecules, thereby enhancing plant resilience to multiple biotic and abiotic stress conditions (Yadav et al. 2017; Dolatabadian 2021).

Numerous bacterial strains have been extensively studied for their capacity to enhance plant growth, with the genera *Azospirillum* and *Bradyrhizobium* standing out due to their ability of nitrogen fixation and, especially in the first genus, of synthesizing phytohormones (Santos et al. 2021; Klepa et al. 2024; Prando et al. 2024; Terra et al. 2025). Plants rely on diverse molecular mechanisms to cope with abiotic stresses, such as heat-shock protein pathways (Wang et al. 2023), and these adaptive responses can be further enhanced by beneficial rhizobacteria that produce protective metabolites. In addition, microbial species such as *Bacillus*, *Chromobacterium*, *Pantoea*, and *Pseudomonas* have also been well-recognized as key contributors to soil fertility and plant health improvement (Ahmad et al. 2012; Burges et al. 2017; Glick and Nascimento 2021; Duchateau et al. 2024; Vasques et al. 2024).

Members of the *Bacillus* genus are widely recognized for their ability to colonize plant roots of various agricultural crops, showing resilience to diverse stress conditions and the ability to suppress a wide range of phytopathogens (Miljaković et al. 2020; Saxena et al. 2020). In addition, these bacteria possess several plant growth-promoting traits, including nutrient solubilization, the synthesis of siderophores and phytohormones, the production of hydrolytic enzymes, among others (Miljaković et al. 2020; Shahid et al. 2021; Vasques et al. 2024). Several species have shown potential for applications in agriculture, for example, *Bacillus velezensis*,* Bacillus altitudinis*, and *Bacillus safensis* (Shan et al. 2024; Vasques et al. 2024; Patel et al. 2025; Risanti et al. 2025; Zhao et al. 2025b).

The genus *Chromobacterium* has also biotechnological potential, mainly related to the synthesis of violacein, a pigmented molecule with antimicrobial, anti-parasitic, anti-cancer, and immunomodulatory properties (Aranda et al. 2011; Wang et al. 2012; Durán, et al., 2016; Ahmed et al. 2021; Sasidharan et al. 2025). The ability to synthesize violacein of *C. violaceum* has also been used as model for studying quorum sensing (QS) (Dimitrova et al. 2023). Some strains have shown PGPB traits by producing molecules with the capacity to suppress soil diseases, solubilizing phosphate, producing siderophores, and acting as biocontrol agents against pests and diseases (Aranda et al. 2011; Wang et al. 2012; Han et al. 2017); however, studies exploring applications in agriculture are still scarce.

The genus *Pantoea* comprises numerous species, reflecting its high genetic and functional diversity (Dahiya et al. 2024). Some members are known to establish beneficial relationships with plants, contributing to plant growth development. Furthermore, due to their diversity and versatility, they can be applied in different sectors of biotechnology, including bioremediation of herbicides and other toxic products (Walterson and Stavrinides 2015). In agriculture, the use of *Pantoea* species as biocontrol agents, such as *Pantoea agglomerans*, has been demonstrated direct antagonism and stimulate plant immunity, which may act either independently or synergistically. Numerous secreted compounds play roles in these mechanisms, including volatile organic compounds (VOCs), antimicrobial compounds, antibiotics, biosurfactants, exopolysaccharide (EPS) and induction of systemic resistance (ISR) in plants (Llontop et al. 2020; Itkina et al. 2022; Duchateau et al. 2024), and there are also species known by the synthesis of phytohormones (Melini et al. 2023). However, there are also pathogenic *Pantoea*, such as *Pantoea ananatis* and *Pantoea stewartii*, which have been associated with grain discoloration and lesions in rice (*Oryza sativa* L.) crops in Africa (Bachabi et al. 2025).

*Pseudomonas* is also well-documented for its plant growth-promoting properties. Reports indicate that the genus can synthesize bio-stimulants and antifungal molecules (Martin-Arjol et al. 2010; García et al. 2022; Mhlongo et al. 2022), induce plant systemic resistance, and facilitate nutrient uptake (Parthasarathy et al. 2025). For example, *Pseudomonas mosselii* and *Pseudomonas koreensis* exhibit the ability to solubilize phosphorus, enhancing its availability to plants. This trait contributes to growth promotion in wheat (*Triticum aestivum* L.), in addition to help in the mitigation of salt and drought stresses in *Arabidopsis thaliana* (Emami et al. 2018; Kalleku et al. 2024).

In previous studies from our group, we have identified six strains at the genus level as *Chromobacterium*, *Pantoea*, *Bacillus*, and *Pseudomonas* with outstanding properties of plant-growth promotion *in vitro* and *in vivo* (Table S1), including phosphate solubilization, biofilm formation, protease and cellulase degradation, indole-3-acetic acid (IAA) production, and exopolysaccharide synthesis (Vasques et al. 2025). Root development in plants is regulated by complex signaling pathways, including transcription factors such as WUSCHEL-related homeobox (WOX) proteins (Abubakar et al. 2023), and can be modulated by microbial phytohormones such as IAA, indole-3-pyruvic acid (IPA), or abscisic acid (ABA) produced by PGPB. The production and modulation of phytohormones by PGPB have a profound impact on plant growth, development, and stress responses. Harnessing the potential of phytohormones-producing PGPB therefore represents a promising strategy for sustainable agriculture, as it reduces reliance on synthetic inputs while promoting environmentally friendly plant growth enhancement (Kejela 2024).

In this context, this study aimed to sequence and characterize the genomes of these six elite strains. Through genomics, the taxonomic classification and the identification of metabolic pathways associated with their plant growth-promoting properties were revealed. Additionally, targeted metabolomic profiling was performed to confirm the production of phytohormones and their precursors molecules by each strain. The results reinforce the potential of these six strains as candidates to compose bio-inputs for agricultural use, contributing to sustainability by replacing chemical inputs.

## Materials and methods

### Bacterial strains

Six strains were used in this study and their information is listed in Table 1. The strains are deposited at the “Diazotrophic and Plant Growth Promoting Bacteria Culture Collection of Embrapa Soja” (WFCC Collection # 1213, WDCM Collection # 1054), in Londrina, State of Paraná, Brazil. For long-term storage, pure cultures of the strains CNPSo 2602, CNPSo 2657, CNPSo 2658, CNPSo 2725 and CNPSo 2799 were cryopreserved in TSB (Tryptone Soya Broth) medium (Atlas et al. 2010; Hungria et al. 2016a), supplemented with 30% (v/v) glycerol, and stored at −80 °C and − 150 °C. The strains were also lyophilized (Hungria et al. 2016b). For strain CNPSo 1954, long-term storage was performed by streaking a pure colony on solid LB (Luria Bertani) medium (Sambrook and Russell 2001) modified, supplemented with 0.5% (w/v) of glycerol, pH 7.5. After bacterial growth, the colonies overlaid with sodium phosphate buffer (Na_2_HPO_4_), and the tubes are sealed and stored at a constant temperature of 28 °C in an angled position of 45°.

**Table 1:** Overview of the studied strains CNPSo 1954, CNPSo 2602, CNPSo 2657, CNPSo 2658, CNPSo 2725, and CNPSo 2799

Strain	Main nomenclature	Secondary nomenclature	Origin	Source of isolation	Isolation references
*Chromobacterium violaceum*	CNPSo 1954	UFAM 16	Manaus-AM, Brazil	Water of the Negro River	Not provided
*Pantoea agglomerans*	CNPSo 2602	IM 01	Sevilha, Spain	Rice	Not provided
*Bacillus velezensis*	CNPSo 2657	PRBS-1 (A3-5)	Londrina-PR, Brazil	Soybean	Cattelan et al. (1998)
*Bacillus altitudinis*	CNPSo 2658	LGMB 178, N2.45	Campo Largo-PR, Brazil	Maize	Ikeda et al. (2013)
*Bacillus safensis*	CNPSo 2725	-	Not provided	Not provided	Not provided
*Pseudomonas* sp.	CNPSo 2799	ET76, IMO 08	Northwestern, Morocco	Rice	Aarab et al. (Aarab, et al., 2016)

### Genomic analysis

#### Bacterial growth conditions

For genome sequencing, each bacterial strain was grown in its optimal growth medium and parameters. *Chromobacterium* sp. strain CNPSo 1954 was grown in 10 mL of liquid LB medium for 24 h, *Pantoea* sp. CNPSo 2602 in modified-YM (Yeast Mannitol) medium (Hungria et al. 2016a) for 72 h, and *Bacillus* spp. strains CNPSo 2657, CNPSo 2658, CNPSo 2725, and *Pseudomonas* sp. CNPSo 2799 in TSB medium for 48 h. All strains were cultivated at 28 °C and 100 rpm.

#### DNA extraction and sequencing

Genomic DNA was obtained using the DNeasy Blood and Tissue kit (Qiagen^®^), according to the manufacturer’s instructions. The quality of the DNA was visualized on a 1% agarose gel under UV light and quantified using a Qubit^®^ device with 1X dsDNA High Sensitivity (HS) kit. Libraries were constructed using the Nextera XT^®^ kit, and genomes were sequenced on the MiSeq (Illumina^®^), as previously described (Megías et al. 2017).

#### Genome analyses

The quality of the sequencing was assessed using the FastQC v0.74 software (Andrews 2010), followed by the removal of adapters and low-quality sequences with the Trimmomatic v0.39.2 software (Bolger et al. 2014). Adapter and other Illumina-specific sequences were removed from the reads using the ILLUMINACLIP step. Subsequently, bases with a quality below the threshold of 25 were removed from the end of the reads, employing a sliding window approach with a quality threshold of 25. Reads with an average quality lower than the threshold of 25 were excluded and reads shorter than 50 bp were discarded. After trimming quality assessment was carried out again with FastQC v0.74 to ensure the reads were properly processed. The genomes assembly was performed using the SPAdes v3.15.5 software using standard parameters (Prjibelski et al. 2020). Contigs smaller than 1,000 bp were filtered and removed from the final assemblies. The quality and contiguity statistics of the assemblies were assessed using QUAST v5.3.0 software (Mikheenko et al. 2023). Genome completeness was assessed with the BUSCO v5.8.0 software (Simão et al. 2015) using the Neisseriales lineage dataset for *Chromobacterium* sp. CNPSo 1954, the Enterobacterales lineage dataset for *Pantoea* sp. CNPSo 2602, and the Bacillales lineage dataset for *Bacillus* spp. CNPSo 2657, CNPSo 2658, and CNPSo 2725. The genome assembly and annotation integrity were evaluated. Whole genome sequencing (WGS) analyses were conducted on the Galaxy Europe online server (usegalaxy.org) (The Galaxy Community 2022). The genome of *Pseudomonas* sp. CNPSo 2799 (= ET76) was originally assembled and analyzed by Aarab and collaborators (2016). In the present study, we employed that assembly and applied an additional filtering step, retaining only contigs higher than 1,000 bp for downstream annotation.

### Phylogenetic inference and taxonomic identification

The genomes of the strains of *Chromobacterium* (TaxID: 535), *Bacillus* (TaxID: 1386), *Pantoea* (TaxID: 53335), and *Pseudomonas* (TaxID: 286) species were obtained from the NCBI RefSeq databases. Average nucleotide identity (ANI) analysis was performed using FastANI v1.13 (Jain et al. 2018) to identify the genomes most closely related to the target strains analyzed in this study (Table S2).

The selected genomes were annotated using PROKKA v1.14.6 (Seemann 2014), generating multifasta protein files. Phylogenomic reconstruction was performed using PhyloPhlAn v3.1.68 (Asnicar et al. 2020), employing the PhyloPhlAn core marker database (2020 version). This database comprises approximately 400 single-copy protein-coding marker genes (http://cmprod1.cibio.unitn.it/databases/PhyloPhlAn/phylophlan_databases.tx). Analyses were individually aligned and concatenated into single amino acid supermatrix.

Maximum likelihood (ML) phylogenetic trees were inferred using IQ-TREE v2.1.5 (Minh et al. 2020). The most suitable substitution model was selected using ModelFinder (Kalyaanamoorthy et al. 2017) implemented in IQ-TREE (-m MFP). Branch support was assessed using 1,000 replicates of ultrafast bootstrap (UFBoot) (Hoang et al. 2018) and 1,000 replicates of the Shimodaira-Hasegawa approximate likelihood ratio test (SH-aLRT) (Guindon et al. 2010).

Phylogenetic trees were visualized using FigTree v1.4.4 and manually edited in Inkscape v1.1 (https://www.inkscape.org/).

#### Average nucleotide identity and DNA-DNA hybridization

Pairwise average nucleotide identity was calculated using OrthoANIu (https://www.ezbiocloud.net/tools/ani) (Yoon et al. 2017). For ANI calculations, the reference genomes of the respective type or reference species were used (GenBank accession numbers in parenthesis): *C. violaceum* ATCC 12,472^T^ (GCF_016890085.1), *P. agglomerans* ATCC 27,155^T^ (GCF_019048385.1), *B. velezensis* FZB42^T^ (GCF_000015785.2), *B. altitudinis* USDA 110^T^ (GCF_001191605.1), *B. safensis* WP8 (GCF_008244765.1), and *Pseudomonas brassicacearum* 51MFCVI2.1 (GCF_000510785.1).

The digital DNA-DNA hybridizations (dDDH) were determined online using the Genome-to-Genome Distance Calculation (GGDC) (https://ggdc.dsmz.de/distcalc2.php) v3.0 (Meier-Kolthoff et al. 2022), using the corresponding reference genome of the species.

### Mining of genomes for antagonism and PGP traits

Gene prediction and functional annotation were performed using PROKKA v1.14.6 software (Seemann 2014), Rapid Annotations using Technology Toolkit (RASTtk) v2.0 subsystems (Brettin et al. 2015) and SEED online server (Overbeek et al. 2013) to group the functions and distributions of predicted genes.

Biosynthetic gene clusters (BGCs) associated with secondary metabolite production were predicted using the antiSMASH v7.0 software (Blin et al. 2023). The tool applies a rule-based framework to detect a wide range of biosynthetic pathways involved in secondary metabolite synthesis. Using the “relaxed” detection strictness setting, antiSMASH identifies both complete clusters, containing all essential biosynthetic components, and partial clusters in which one or more functional elements may be absent. With the antiSMASH predictions, which indicate the start and end coordinates of each BGCs, we were able to calculate the size of the biosynthetic clusters and, consequently, determine the proportion of the genome dedicated to BGCs involved in secondary metabolite production.

The functional annotation of genomes was performed with the EggNOG-mapper (Huerta-Cepas et al. 2019). Kyoto Encyclopedia of Genes and Genomes (KEGG) database through BLASTp (Kanehisa et al. 2022) annotations were derived from the EggNOG-mapper results.

Antibiotic resistance was predicted using the Resistance Gene Identifier (RGI) tool available in the Comprehensive Antibiotic Resistance Database (CARD) (http://card.mcmaster.ca) (Alcock et al. 2023). Antibiotics were considered as showing resistance when the analysis returned an identity value equal to or greater than 90% and coverage equal to or greater than 80%.

CAZymes were annotated through a search in the dbCAN3 server databases, with a combination of dbCAN, DIAMOND and HMMER (CAZyme family and CAZyme subfamilies) tools (Zheng et al. 2023). Only CAZymes identified by all three tools were retained for analysis. In our study, we focused on the classes of enzymes that can promote plant protection by degrading fungal cell walls. These include six enzyme classes: glycoside hydrolases (GHs); glycosyltransferases (GTs); polysaccharide lyases (PLs); carbohydrate esterases (CEs); auxiliary activities (AAs); and carbohydrate-binding modules (CBMs).

#### Bacterial secretion and CRISPR-Cas systems

The MacsyFinder v2.1 (Abby et al. 2014) tool with default settings and hmm TXSS profiles were used to annotate the secretion systems and CRISPR-Cas Systems presented in the bacteria strains.

### Targeted metabolomics of PGPB

#### Preparation of pre-inocula and cultures of PGPB

For all PGPB strains, except for *Chromobacterium* sp. CNPSo 1954, pre-inocula were prepared by inoculating 30 µL of cryopreserved samples into 10 mL of culture media contained in 25 mL schott bottles; culture media for each strain were as describe in Bacterial growth conditions, and bacteria were grown for 24 h (*Bacillus* CNPSo 2657 and CNPSo 2725), 48 h (B*acillus* CNPSo 2658, *Pseudomonas* CNPSo 2799), or 72 h (*Pantoea* CNPSo 2602). Cultures were prepared by inoculating 1 mL of each pre-inoculum into corresponding 100 mL of culture medium in 250 mL schott bottles. Pre-cultures and cultures were grown at 28 °C and 100 rpm.

The pre-inoculum for *Chromobacterium* sp. CNPSo 1954, was prepared from preserved cultures maintained on inclined tubes containing solid LB medium supplemented with 0.5% of glucose. To enhance preservation, 0.1 M phosphate buffer was added over the culture surface, which partially solubilized the culture and resulted in a suspension used as the inoculum source. A 30 µL aliquot of this suspension was transferred to 50 mL Erlenmeyer flasks containing 10 mL of TSB and modified-TSB (glucose-free), adapted from Ahmad et al. (2012) (17.0 g L⁻¹ casein, 3.0 g L⁻¹ soybean meal peptone, 5.0 g L⁻¹ NaCl, 2.5 g L⁻¹ K₂HPO₄; pH 7.0). After initial growth, 1 mL of the culture was transferred to 500 mL Erlenmeyer flasks containing 100 mL of the respective media. The flasks were sealed with gauze and hydrophobic cotton, and bacteria were always grown at 30 °C for 48 h and 200 rpm.

All bacteria were grown in three biological replicates in addition to non-inoculated triplicated controls grown under the same conditions.

#### Isolation of cell-free metabolites (CFMs)

After the incubation period, all cultures of PGPB were aliquoted into 50 mL Falcon tubes, acidified with 1% formic acid (85%), and manually homogenized for 2 min to ensure uniform mixing. The samples were then subjected to centrifugation at 9500 rpm for 10 min at 4 °C. The supernatants were carefully transferred into new 50 mL Falcon tubes, while the cell pellets were discarded. To further ensure the removal of any remaining cells, a second centrifugation was performed under identical conditions.

The CFMs were filtered through sterile 0.22 μm hydrophilic PVDF membranes (Millex-GV^®^, Merck Millipore, Ireland). To verify the efficiency of cell removal, 100 µL aliquots of the CFMs were plated by spread plating, in duplicate, onto the specific culture media used for each strain, supplemented with 12 g L⁻¹ of bacteriological agar. These plates were incubated under the same time and temperature conditions specified for culture preparation.

Following incubation and confirmation of complete cell removal, the samples were stored in 50 mL Falcon tubes at −80 °C until further analysis.

The non-inoculated controls corresponding to each cultivation condition were subjected to the same protocol for obtaining CFMs. This standardization ensured that the control samples were identical to their respective cultures in all procedures, including medium composition, acidification, homogenization, centrifugation, filtration, and storage, differing solely by the absence of the bacteria.

#### Solid-phase extraction (SPE)

Following this initial preparation, aliquots of the CFMs and controls were subjected to solid-phase extraction (SPE) for the concentration and purification of analytes, making the metabolites suitable for subsequent high-sensitivity analyses (Rodrigues et al. 2009). The SPE protocol was specifically optimized to efficiently isolate phytohormones, reducing potential contaminants that could interfere with subsequent Reverse Phase Ultra Performance Liquid Chromatography coupled with Photodiode Array (RP-UPLC-PDA) analysis.

Solid-phase extraction was conducted using an SPE-ED Mate Glass Vacuum Manifold system (without vacuum) with C18-E Strata columns (0.5 g per 0.006 L, Phenomenex). The extraction protocol was adapted from Rodrigues et al. (2009) and involved the following five sequential steps: (1) Cartridge Conditioning: The C18 cartridges were conditioned with 20 mL of methanol (MeOH) (LC-MS grade, Sigma Aldrich, Taufkirchen, Germany) to activate the stationary phase; (2) Cartridge Equilibration: The cartridges were equilibrated with 10 mL of ultrapure water containing 1% formic acid (85% purity, P.A.) to ensure optimal retention of analytes; (3) Sample Loading: Acidified samples (20 mL, with 1% formic acid) were carefully loaded onto the conditioned cartridges to allow for effective adsorption of target compounds; (4) Cartridge Washing: The cartridges were washed with 10 mL of ultrapure water containing 1% formic acid to remove impurities while retaining the analytes; (5) Analyte Elution: The retained analytes were eluted using 10 mL of MeOH.

After the SPE process, the eluates were dried using a Dry Block system under a gentle nitrogen stream, without applying heat, to prevent degradation of thermolabile compounds. Once dried, the samples were reconstituted in 1 mL of a water-acetonitrile (H_2_O-ACN) solution (1:1, v/v). For optimal resuspension, the samples were vortexed for 1 min and subjected to an ultrasonic bath for 20 min at room temperature.

This method was designed to ensure efficient purification and concentration of analytes, making the extracts suitable for subsequent high-sensitivity analyses.

#### Method validation for phytohormone analysis in microbial cultures

The validation of the method for assessing phytohormones in microbial cultures was conducted based on the Eurachem/CITAC Guide (Barwick 2016), adopted as the primary framework for this study. This guide is widely recognized for its applicability to compound analysis in complex matrices, such as bacterial culture media. The validation strategy incorporated key performance parameters: calibration curve linearity (R²), limits of detection (LOD) and quantification (LOQ), accuracy (expressed as recovery %), precision (as relative standard deviation – RSD%), dynamic linear range, and matrix effects.

To support the validation process, stock solutions of a comprehensive panel of phytohormones and precursors molecules were prepared in methanol at 200 mg L^− 1^, including: 2,4-dihydroxybenzoic acid (2,4-DHBA), abscisic acid (ABA), cis-jasmone (CJ), DL-indole-3-lactic acid (ILA), gibberellic acid (GA), indole-3-acetamide (IAM), indole-3-acetic acid (IAA), indole-3-acetonitrile (IAN), indole-3-butyric acid (IBA), indole-3-pyruvic acid (IPA), (±) L-tryptophan (TRP), methyl salicylate (MeSA), salicylic acid (SA), and trans-cinnamic acid (*t*-CA). Calibration curves were also constructed using DYGS culture media (Fukami et al. 2018) with final concentrations ranging from 0.1 to 10 µg mL⁻¹. Linearity was evaluated across eight concentration levels, each injected in septuplicate. Chromatographic analyses were performed using a RP-UPLC-PDA and calibration curves were obtained by linear regression of peak areas data (Table S3). The runs were conducted on UPLC H-Class system (Waters Corporation, USA) equipped with an Acquity UPLC HSS C18 SB reverse-phase column (100 mm x 2.1 mm × 1.8 μm), maintained at 40 °C. The mobile phase consisted of I (Phase A) and ultrapure water (Phase B), both containing 0.1% formic acid, with a flow rate of 0.4 mL min^− 1^. The gradient used for the separation is detailed in the supplementary Table S4. LOD and LOQ were defined based on signal-to-noise (S/N) ratios of ≥ 3 and ≥ 10, respectively.

Accuracy and precision were evaluated through spike-and-recovery experiments. Dextrose yeast glucose sucrose (DYGS) medium was spiked with known concentrations (1.0, 2.5 and 5.00 µg mL⁻¹) of for representative hormones, indole-3-acetamide (IAM), abscisic acid (ABA), indole-3-butiric acid (IBA), and indole-3-acetic acid (IAA). These metabolites were selected for evaluating method accuracy and precision because they represent distinct yet relevant chemical classes within the target phytohormone panel, including indole-derived and non-indole phytohormones. Collectively, they cover a representative range pf physicochemical properties (polarity), detection features (UV absorbance), and chromatographic behavior (retention times). In addition, the selected analytes encompass the main biosynthetic routes investigated in this study (IAA, IPA, IBA and ABA), which are directly related to plant growth-promotion traits inferred from genome mining. Method validation was conducted in accordance with internationally recognized recommendations (Eurachem/CITAC Guide; Barwick 2016), using the established extraction protocol followed by triplicate analysis (Fig. S1, Table S5). Repeatability was considered acceptable, as all compounds presented relative standard deviation (RSD, %) below 15%.

#### Phytohormone profiling and quantification

CFM and control sample analyses were performed using the same RP-UPLC-PDA method and chromatographic conditions as in method validation. All system operations, data acquisition, and analysis are managed using the Empower™ 3 (Waters Corporation, build 3471, copyright 2010; base package, system suitability).

To stabilize the column, ten injections of 3 µL of a H_2_O-I solution (9:1, v/v) were performed. This was followed by the injection of 3 µL aliquot of a standard mixture at 5 mg L^− 1^, comprising all compounds listed in Sect. 2.5.4. Sample injections were then carried out, with an additional standard injection performed after every ten samples to ensure system stability and calibration consistency.

The PDA detector was set to scan absorbance in the range of 190–400 nm. Each analyte was identified by matching its retention time and UV spectrum to those of authentic standards. Quantification was achieved by interpolating the peak areas against the linear calibration curves.

Statistical analyses were conducted using biological replicates as the experimental units and technical replicates were performed exclusively to assess analytical repeatability. Regarding the homoscedasticity and normality were assessed using the Shapiro-Wilk test and Levene’s test, respectively. When a phytohormone or associated compounds were detected in more than one CFMs sample, a univariate analysis of variance (ANOVA) was performed using a completely randomized design, followed by Tukey’s pos hoc test (*p* ≤ 0.05). For tryptophan, which was detected in the CFMs and control samples and did not meet the assumptions for parametric analysis assessed by ANOVA, comparisons between CFMs and their respective controls were made using non-parametric Wilcoxon test (*p* ≤ 0.05). All statistical analyses were performed using the R programming language (R Core Team 2023) in the RStudio environment, with the ExpDes.pt package (Ferreira et al. 2021).

## Results

### Genome assemblies of bacteria growth-promoting

Our *de novo* assemblies of the bacterial species (Table 2) resulted in genomes ranging from 3,792,767 bp in *Bacillus* sp. CNPSo 2658 to 6,681,652 bp in *Pseudomonas* sp. CNPSo 2799. The G + C content was highest in *Chromobacterium* sp. CNPSo 1954, with 65.10 mol%, and lowest in *Bacillus* sp. CNPSo 2658, 41.39 mol%.

**Table 2:** General characteristics of the genome of the plant growth-promoting bacteria of this study

Genome statistics	*Chromobacterium violaceum*	*Pantoea agglomerans*	*Bacillus velezensis*	*Bacillus altitudinis*	*Bacillus safensis*	*Pseudomonas sp.*
	CNPSo 1954	CNPSo 2602	CNPSo 2657	CNPSo 2658	CNPSo 2725	CNPSo 2799
Size (bp)	4,797,272	4,935,403	4,237,140	3,792,767	3,979,002	6,681,652
G + C content (mol %)	65.10	55.08	45.95	41.39	41.72	60.69
Number of contigs	251	282	531	339	798	68
Number of contig > = 1000 bp	78	79	37	60	67	55
L50^a^	12	14	5	9	11	7
N50^b^ (bp)	120,898	105,863	240,505	160,418	116,87	306,697
Genome completeness (%)	99.0	99.5	99.6	99.6	100.0	99.9
Genome coverage (x)	270	128	436	258	259	27
Accession number	JBLZSQ000000000	JBLZSP000000000	JBLZSO000000000	JBLZSN000000000	JBLZSM000000000	LNAB00000000
CDS^c, d^	4,689	4,720	4,333	3,905	4,026	6,078
RNAs^d^	48	68	28	64	61	66
Features in Subsystem^d^	1676	1805	1624	1562	1589	2460
% of BGCs in the genome	4.45	4.26	18.03	8.30	8.28	12.32

^a^Minimum number of contigs comprising the largest half of the genome

^b^The length such that half of all sequence is in contigs of this size or larger

^c^Coding sequences (CDS)

^d^Analysis obtained through RAST software

Genome completeness levels assessed using BUSCO indicated that the assemblies were successful, ranging from 100% in *Bacillus* sp. CNPSo 2725 to 99% in *Chromobacterium* sp. CNPSo 1954 (Table 2). The genomes were deposited at the GenBank database and their accession numbers are shown in Table 2.

Genome comparisons of *Chromobacterium* sp. CNPSo 1954, *Pantoea* sp. CNPSo 2602, *Bacillus* spp. CNPSo 2657, CNPSo 2658, CNPSo 2725, and *Pseudomonas* sp. CNPSo 2799 revealed ANI values of 98.7%, 98.6%, 98.9%, 98.1%, 96.4%, and 80.8%, respectively, when compared to the reference genomes of the closest type species (Table S6). Corresponding dDDH values were 87.8%, 88.6%, 90.6%, 83.5%, 69.4%, and 24.6%, respectively. Based on the accepted species delineation thresholds of 95–96% for ANI and 70% for dDDH (Goris et al. 2007; Helene et al. 2022), the first five strains had their species defined as *C. violaceum* CNPSo 1954, *P. agglomerans* CNPSo 2602, *B. velezensis* CNPSo 2657, *B. altitudinis*, CNPSo 2658, and *B. safensis* CNPSo 2725, while for *Pseudomonas* sp. CNPSo 2799, a putative novel species, phylogenetic analyses revealed that is closely related to the *Pseudomonas fluorescens* complex. Phylogenetic trees for all six strains were obtained and are shown as supplementary figures (Figs. S2 to S5).

#### Gene prediction and functional annotation of bacteria genomes

The genome analysis of the six bacterial strains showcased a range of coding sequences (CDS), ranging from 3,905 in *B. altitudinis* CNPSo 2658 to 6,078 in *Pseudomonas* sp. CNPSo 2799, and RNAs ranging from 28 in *B. velezensis* CNPSo 2657 to 68 in *P. agglomerans* CNPSo 2602 (Table 2).

Using the clusters of orthologous groups in the RAST online server, the coding sequences were grouped into 28 categories (Fig. S6) and the numbers of genes annotated for each bacterial strain were as follows: 1,676 for *C. violaceum* CNPSo 1954; 1,805 for *P. agglomerans* CNPSo 2602; 1,624 for *B. velezensis* CNPSo 2657; 1,562 for *B. altitudinis* CNPSo 2658; 1,589 for *B. safensis* CNPSo 2725; and 2,460 for *Pseudomonas* sp. CNPSo 2799. The genes were classified into cellular components, molecular functions, and biological processes. The most prominent functional category, with the highest number of genes across all strains, was the amino acids and derivatives, ranging from 278 genes in *B. altitudinis* CNPSo 2658 to 475 in *Pseudomonas* sp. CNPSo 2799. In contrast, the categories of nodulation and photosynthesis were not represented in any of the analyzed genomes.

#### Bacterial secretion system

The three *Bacillus* strains, *B. velezensis* CNPSo 2657, *B. altitudinis* CNPSo 2658, and *B. safensis* CNPSo 2725, share a complete repertoire of genes involved in genetic competence, from the recognition of extracellular DNA to its translocation and regulation by ComK (Table S7), a master transcriptional regulator that controls the expression of genes required for DNA uptake and integration (Jakobs and Meinhardt 2015). Genomic analyses showed that *C. violaceum* CNPSo 1954 presents a greater secretion repertoire than the other strains, with secretion systems from T1SS to T6SS. Strain *P. agglomerans* CNPSo 2602 has the T1SS, T4SS, T5SS, and T6SS. *Pseudomonas* sp. CNPSo 2799 presents all bacterial secretion nanomachines, including the Tad/Flp system, except for the T3SS (Table S7).

#### CRISPR-Cas system

CRISPR-Cas systems enable genome editing by generating specific double strand breaks in DNA. Among the strains, only *P. agglomerans* CNPSo 2602 harbors a Class 1, subtype I-E CRISPR-Cas system, composed of the genes *cas1*, *cas2*, *cas3*, *cas5*, *cas6*, *cas7*, *cas8e*, *cse2gr11*, and multiple copies of proteins with the DEDDh domain (Table S8).

### Genome mining for antagonism, immunity enhancing traits and plant growth promoting in bacteria genomes

#### Secondary metabolite biosynthetic gene clusters

The genome of *C. violaceum* CNPSo 1954 revealed BGCs in 4.45% of the genome (Table 3, Table S9). There are at least 10 BGCs, including the non-ribosomal peptide synthetases (NRPS), hydrogen-cyanide, indole, and NRP-metallophore, with high similarity to viobactin, violacein and chromobactin. Other clusters, beralactone, terpene, and RiPP-like showed low similarity (< 40%), indicating potential novelty.

For the genome of *P. agglomerans* CNPSo 2602, the antiSMASH analysis identified BGCs accounting for 4.26% of the genome (Table 3, Table S9), with the presence of at least seven BGCs, among then some with high similarity to frederiksenibactin, desferrioxamine E, carotenoid, and arylpolyenes. Additional clusters redox-cofactor, RiPP-like and hserlactone showed low or no similarity.

The BGCs of *B. velezensis* CNPSo 2657 comprised 18.03% of the genome (Table 3, Table S9), with the presence of at least 20 clusters, some with high similarity to bacilysin, macrolactin H, bacillibactin, and fengycin. Clusters resembling difficidin and bacillaene were also identified, along with several with low similarity, suggesting high metabolic diversity.

*B. altitudinis* CNPSo 2658 contained BGCs representing 8.30% of the genome (Table 3, Table S9), with the presence of at least 10 BGCs, with two clusters showing high similarity to bacilysin and lichenysin. Other clusters NI-siderophore, betalactone, and NRPS-like showed moderate or low similarity, and several did not match any known cluster.

*B. safensis* strain CNPSo 2725 has 8.28% of its genome accounted for BGCs, but only one cluster, NRP-metallophore and NRPS, showed high similarity to a known cluster, related to the synthesis of bacillibactin, bacillibactin E and F (Table 3, Table S9). Additional clusters showed partial similarity to clusters schizokinen, fengycin, bacilysin, and lichenysin, as well as others with no known correspondence.

In the genome of *Pseudomonas* sp. CNPSo 2799, 12.32% of the total genomic content is dedicated to BGCs involved in secondary metabolite production (Table 3, Table S9), with the presence of at least 33 BGCs, the largest repertoire identified. They included 14 clusters showing high similarity to compounds such as 2,4-diacetylphloroglucinol histicorrugatin, corpeptin, nunapeptin, and kolossin. Several others exhibited low or no similarity, indicating strong potential for novel metabolites.

**Table 3:** Biosynthetic gene clusters class involved in the production of secondary metabolites in bacteria growth-promoting genomes

Most similar known cluster	*Chromobacterium violaceum*	*Pantoea agglomerans*	*Bacillus velezensis*	*Bacillus altitudinis*	*Bacillus safensis*	*Pseudomonas sp.*
Class	CNPSo 1954	CNPSo 2602	CNPSo 2657	CNPSo 2658	CNPSo 2725	CNPSo 2799
Alkaloid	+	-	-	-	-	+
NRP	+++	+	+++++	+	++	++++++++++++++++
NRP: Cyclic depsipeptide	-	-	-	-	-	+
NRP: Lipopeptide	+	-	+++	-	-	++
NRP: Lipopeptide + Saccharide: Hybride/tailoring Saccharide	-	-	-	-	-	+
NRP + Polyketide	-	+	-	+	-	+
Other	++	++	+	+++	+++++	++
Polyketide	-	-	++++	-	-	+
Polyketide + NRP	-	-	++	-	-	-
Saccharide	-	-	+	-	-	-
Terpene	-	+	-	-	-	-

(+) refers to the presence of the gene, and how many times it appears and the number of copies of the gene in the genome

(-) refers to the absence of the gene clusters in the genome

#### Antibiotic resistance and antimicrobial compounds

**Fig. 1:**
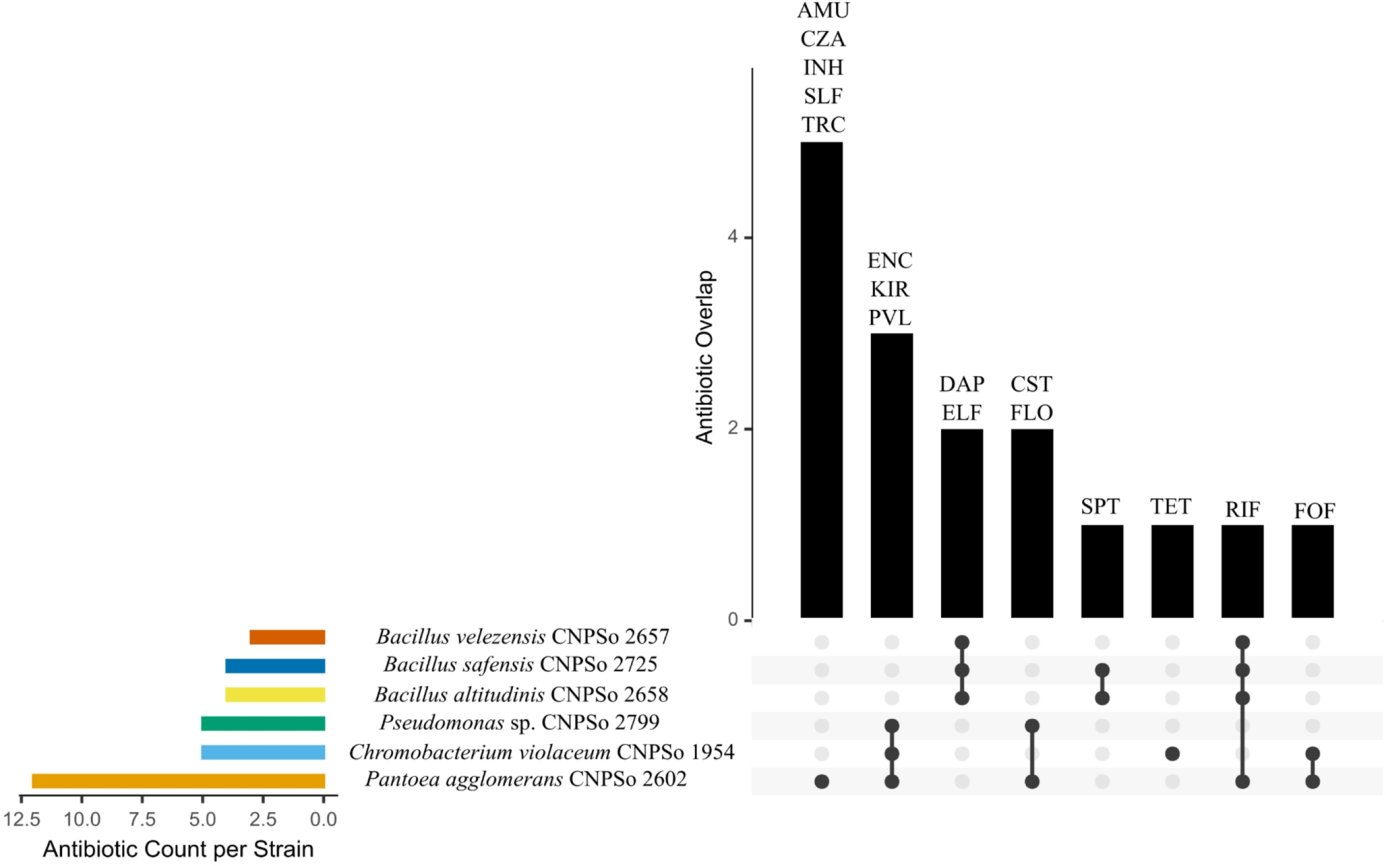
Comparative antibiotic resistance profiles of bacterial strains CNPSo 1954, CNPSo 2602, CNPSo 2657, CNPSo 2658, CNPSo 2725, and CNPSo 2799, highlighting shared and unique resistance traits among strains, as identified using the Comprehensive Antibiotic Resistance Database (CARD- https://card.mcmaster.ca). AMU: aminocoumarin; CZA: ceftazidime-avibactam; CST: colistin; DAP: daptomycin; ELF: elfamycin; ENC: enacyloxin IIa; FLO: fluoroquinolones; FOF: fosfomycin; INH: isoniazid; KIR: kirromycin; PLV: pulvomycin; RIF: rifampin; SPT: spectinomycin; SLF: sulfonamides; TET: tetracycline; and TRC: triclosan

*In silico*-based analysis of antibiotic resistance profile indicated that *P. agglomerans* CNPSo 2602 exhibited the most diverse repertoire of resistance genes, presenting 12 distinct classes of antibiotics (Fig. 1, Table S10). In contrast, *B. velezensis* CNPSo 2657 exhibited the most restricted set, showing resistance to three antibiotics. *B. altitudinis* CNPSo 2658 and *B. safensis* CNPSo 2725 showed resistance to four antibiotics. *C. violaceum* CNPSo 1954 and *Pseudomonas* sp. CNPSo 2799 showed resistance to five antibiotics each.

Regarding the biosynthesis of antimicrobial compounds, the three *Bacillus* strains were primarily associated with this trait, as evidenced by the presence of operons such as *aus* and *bac* in their genomes (Fig. S7 to S9).

#### Carbohydrate-active enzymes

Regarding CAZymes, an average of 79 CAZymes were detected in the complete genomes of the strains (Table 4). The genome with the highest number of CAZymes was identified in *P. agglomerans* CNPSo 2602, which contained 93 CAZymes, whereas the the lowest was in *C. violaceum* CNPSo 1954, with 66 CAZymes. The most prominent CAZymes in all strains were glycoside hydrolases and glycosyltransferases, representing more than 50.7% of the CAZymes repertoire, while carbohydrate esterases, carbohydrate binding modules, polysaccharide lyases and auxiliary activities were less frequent. The bacteria *B. velezensis* CNPSo 2657 and *Pseudomonas* sp. CNPSo 2799 exhibited CAZymes across all classes. In contrast, *C. violaceum* CNPSo 1954 lacked CAZymes in the polysaccharide lyases and carbohydrate-binding modules classes, and *P. agglomerans* CNPSo 2602 did not present for the polysaccharide lyases class. Finally, *B. altitudinis* CNPSo 2658 and *B. safensis* CNPSo 2725 showed no CAZymes associated with the auxiliary activities class.

**Table 4:** Distribution of CAZymes in bacteria strains CNPSo 1954, CNPSo 2602, CNPSo 2657, CNPSo 2658, CNPSo 2725, and CNPSo 2799 genomes

	*C. violaceum*	*P. agglomerans*	*B. velezensis*	*B. altitudinis*	*B. safensis*	*Pseudomonas sp.*
	CNPSo 1954	CNPSo 2602	CNPSo 2657	CNPSo 2658	CNPSo 2725	CNPSo 2799
Glycoside Hydrolases (GHs)	23	40	44	32	37	22
Glycosyltransferases (GTs)	32	47	32	27	28	34
Polysaccharide Lyases (PLs)	0	0	3	2	2	4
Carbohydrate Esterases (CEs)	6	2	9	14	13	3
Auxiliary Activities (AAs)	5	1	1	0	0	2
Carbohydrate-binding modules (CBM)	0	3	3	2	2	2
TOTAL	66	93	92	77	82	67

#### Metabolic pathways related to putative plant growth-promoting properties

Focusing on the predicted metabolic pathways related to plant growth-promotion, *Pseudomonas* sp. CNPSo 2799 exhibited the highest number of annotated pathways, 270. *B. safensis* CNPSo 2725, *B. altitudinis* CNPSo 2658, and *B. velezensis* CNPSo 2657 exhibited fewer annotated pathways, with 235, 236, and 251 annotations, respectively, while *C. violaceum* CNPSo 1954 and *P. agglomerans* CNPSo 2602 showed 266 and 261 annotated pathways, respectively.

##### Nitrogen metabolism

Strains *C. violaceum* CNPSo 1954 (Fig. S10), *P. agglomerans* CNPSo 2602 (Fig. S11), *B. velezensis* CNPSo 2657 (Fig. S12), and *Pseudomonas* sp. CNPSo 2799 (Fig. S13), exhibited enriched nitrogen metabolism pathways, with all four harboring the operons nitrate reductase/nitrite oxidoreductase, alpha subunit (*nar*), assimilatory nitrate reductase catalytic subunit (*nas*), and nitrite reductase (*nir).* Additionally, *C. violaceum* CNPSo 1954, *P. agglomerans* CNPSo 2602 and *B. velezensis* CNPSo 2657 also carry the operon nitrate reductase/nitrite oxidoreductase, alpha subunit (*nxr*). The MFS transporter, NNP family, nitrate/nitrite transporter (*nrt*) operon was identified in *C. violaceum* CNPSo 1954, *P. agglomerans* CNPSo 2602, and *Pseudomonas* sp. CNPSo 2799. Both *P. agglomerans* CNPSo 2602 and *Pseudomonas* sp. CNPSo 2799 harbor the gene nitric oxide reductase subunit B (*nor*), whereas *P. agglomerans* CNPSo 2602 uniquely possesses nitrate reductase (cytochrome) (*nap*) and *nir.* Finally, *Pseudomonas* sp. CNPSo 2799 was the only strain to carry *nir*, and nitrous-oxide reductase (*nos*) genes.

##### Biofilm formation

*C. violaceum* CNPSo 1954, *P. agglomerans* CNPSo 2602, and *Pseudomonas* sp. CNPSo 2799 showed a prominent presence of genes associated with biofilm formation (Figs. S14 to S16). Among them, there were genes belonging to a variety of operons, including c-di-GMP phosphodiesterase (*adr*), two-component system, OmpR family, aerobic respiration control sensor histidine kinase ArcB (*arc*), chemosensory pili system protein ChpA (sensor histidine kinase/response regulator) (*chp*), flagellar transcriptional activator FlhD (*flh*), NA polymerase sigma factor FliA (*fli*), two-component system, response regulator FlrC (*flr*), two-component system, NarL family, sensor histidine kinase BarA (*gac*), LysR family transcriptional regulator, glycine cleavage system transcriptional activator (*gcv*), glucose-1-phosphate adenylyltransferase (*glg*), two-component system, HptB-dependent secretion and biofilm response regulator (*hsb*), biofilm PGA synthesis protein PgaA (*pga*), anthranilate synthase component II (*phn*), twitching motility protein PilJ (*pil*), two-component system, NarL family, capsular synthesis sensor histidine kinase RcsC (*rcs*), rhamnosyltransferase subunit A (*rhl*), RNA polymerase sigma-54 factor (*rpo*), two-component system, NarL family, sensor histidine kinase BarA (*var*), diguanylate cyclase (*yeg*), and polysaccharide biosynthesis/export protein (*wsp*).

##### Siderophore production

*P. agglomerans* CNPSo 2602, *C. violaceum* CNPSo 1954, *B. velezensis* CNPSo 2657, *B. safensis* CNPSo 2725, and *Pseudomonas* sp. CNPSo 2799 exhibited metabolic pathways related to siderophore biosynthesis (Figs. S17 to S21), including genes such as 2,3-dihydroxybenzoate (aryl-carrier protein) ligase (*dbb* and *vib*). The *Bacillus* strains also harbor *eat* and *mxc*, while *C. violaceum* CNPSo 1954 and *P. agglomerans* CNPSo 2602 possess siderophore-related genes such as L-serine (L-seryl-carrier) protein ligase *(ent*) and 2,3-dihydroxybenzoate (aryl-carrier protein) ligase *(mxc).*

##### Phosphate solubilization

Regarding phosphonate and phosphinate metabolism pathways, which are associated with phosphate solubilization pathways, the strains *C. violaceum* CNPSo 1954 and *P. agglomerans* CNPSo 2602 exhibited prominence, with eight and seven associated pathways, respectively (Figs. S22, S23).

##### Quorum sensing

As expected, *C. violaceum* CNPSo 1954 exhibited genetic features associated with quorum sensing mechanisms, including operons such as tagatose 1,6-diphosphate aldolase (*lac*), anthranilate synthase component I (*phn*), acyl homoserine lactone synthase (*phz*), two-component system, OmpR family, response regulator QseB (*qse*), GTP cyclohydrolase II (t*ox*) and tryptophan oxidase VioA (*vio*) (Fig. S24).

##### Tryptophan biosynthesis

**Fig. 2:**
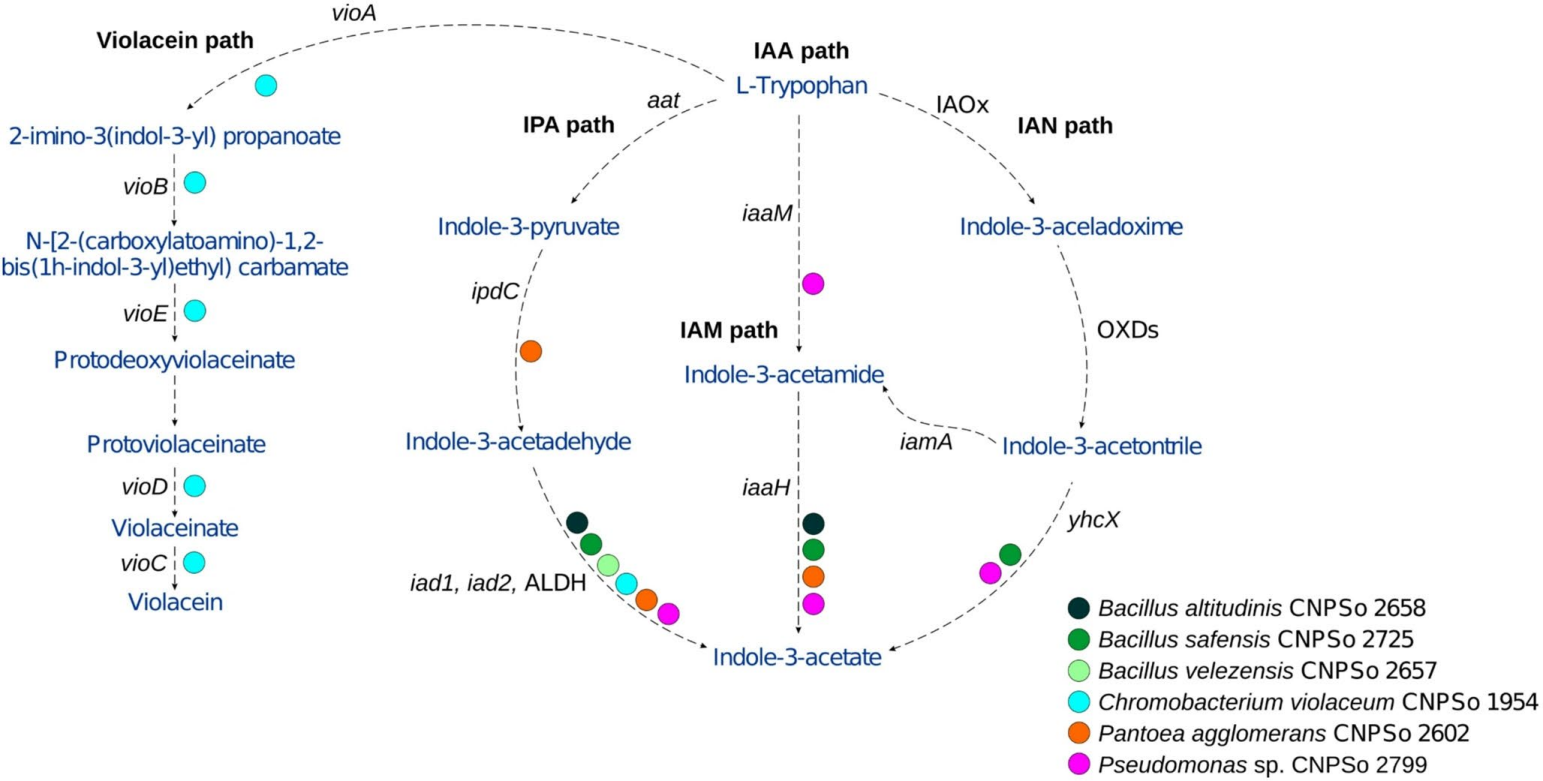
Gene analysis of violacein and indole-3-acetic acid (IAA) biosynthetic pathways with six plant growth-promoting bacteria. Dashed arrows show metabolic flux from L-tryptophan (TRP)

All six strains harbor genes associated with the tryptophan biosynthesis pathway and the operon tryptophan synthase (*trp*) (Fig. 2; Table 5, Figs. S25 to S30). The three *Bacillus* also contain the NAD-dependent aldehyde dehydrogenase (*aldH*) gene. *Pseudomonas* sp. CNPSo 2799 carries both *aldH* and tryptophan-2-monooxygenase (*iaaM*), whereas *P. agglomerans* CNPSo 2602 harbors indole-3-pyruvate decarboxylase (*ipdC*) and indole-3-acetamide hydrolase (*iaaH*). With respect to IPA pathway, the aspartate aminotransferase (*aspC*) gene is present in *P. agglomerans* CNPSo 2602, *B. velezensis* CNPSo 2657, and *Pseudomonas* sp. CNPSo 2799, while *P. agglomerans* CNPSo 2602 also possesses the *ipdC* gene. Additionally, *Pseudomonas* sp. CNPSo 2799 contains the *iaaM* gene, important for the IBA pathway. All six strains possess the 1-deoxy-D-xylulose-5-phosphate synthase (*dxs*) gene, which is associated with ABA biosynthesis.

**Table 5:** Detection of phytohormones and precursors molecules in cell-free metabolites (CFMs) of six potential plant growth-promoting bacterial (PGPB) strains and the corresponding biosynthetic genes

Compounds	Acronym	*C. violaceum*		*P. agglomerans*	*B. velezensis*	*B. altitudinis*	*B. safensis*	*Pseudomonas* sp.
		CNPSo 1954		CNPSo 2602	CNPSo 2657	CNPSo 2658	CNPSo 2725	CNPSo 2799
		Unmodified media	Modified media					
2,4-Dihydroxybenzoic acid	2,4-DHBA	-	-	-	-	-	-	-
		*entC*,* menF*		*entC*	*dhbC*,* menF*	-	*dhbC*	*pobA*
Abscisic acid	ABA	-	-	-	+	-	-	-
		*dxs*		*crtB*,* crtI*,* crtY*,* dxs*,* idi*	*dxs*	*dxs*,* idi*	*dxs*,* idi*	*dxs*
Cis-jasmone	CJ	-	-	-	-	-	-	-
		*fadL*,* fadD*		*fadL*,* fadD*	*fadD*	*-*	*fadD*	*fadD*,* fadL*
DL-indole-3-lactic acid	ILA	-	-	-	-	-	-	-
		*feoB*		*feoB*,* ipdC*	*aldH*,* ldh*	*aldH*,* ldh*	*aldH*,* ldh*	*aldH*,* ldh*
Indole-3-acetic acid	IAA	-	+	+	+	+	+	+
		*trpC*		*iaaH*,* ipdC*,* trpC*	*aldH*,* trpC*	*aldH*,* trpC*	*aldH*,* trpC*	*aldH*,* iaaM*,* trpC*
Indole-3-acetonitrile	IAN	-	-	-	-	-	-	-
		*asnB*		*asnB*	*asnB*	*asnB*	*asnB*	*asnB*
Indole-3-butyric acid	IBA	-	-	-	+	+	+	+
		*tyrB*		*aspC*,* iaaH*,* ipdC*,* tyrB*	*aldH*,* aspC*	*aldH*	*aldH*	*aldH*,* aspC*,* iaaM*,* tdcB*,* tyrB*
Indole-3-pyruvic acid	IPA	+	+	-	-	-	+	-
		*trpA*,* trpB*,* tyrB*		*aspC*,* ipdC*,* trpA*,* trpB*,* tyrB*	*aspC*,* trpA*,* trpB*	*trpA*,* trpB*	*trpA*,* trpB*	*aspC*,* trpA*,* trpB*,* tyrB*
L-tryptophan	TRP	+	+	-	+	+	+	+
		*trpA*,* trpB*,* trpC*,* trpD*,* trpE*,* trpF*,* trpG*		*trpA*,* trpB*,* trpC*,* trpD*,* trpE*,* trpG*,* trpR*	*trpA*,* trpB*,* trpC*,* trpD*,* trpE*,* trpF*,* trpP*	*trpA*,* trpB*,* trpC*,* trpD*,* trpE*,* trpF*,* trpP*	*trpA*,* trpB*,* trpC*,* trpD*,* trpE*,* trpF*,* trpP*	*trpA*,* trpB*,* trpC*,* trpD*,* trpE*,* trpF*,* trpG*,* trpI*
Salicylic acid	SA	-	-	-	-	-	-	-
		*entC*,* menF*,* phzF*		*entC*,* phzF*	*pchA*	*pchA*	*pchA*	*pchA*,* pchB*,* phzF*
*Tran*s-cinnamic acid	*t*-CA	+	-	-	-	-	-	-
		*pal*,* tyrB*		*aspC*,* pal*,* tyrB*	*aspC*	-	-	*aspC*,* paa*,* pal*,* tyrB*

Methyl salicylate (MeSA), and indole-3-acetamide (IAM) were not included in the table, as no data were found for these compounds in either the metabolomic or genomic analysis. For each strain (columns), the first row presents the metabolomic data: (+) indicates compounds detected and (-)

### Metabolomic insights into phytohormone biosynthesis by PBPG

Among the 14 metabolites evaluated across the six elite strains, five were detected in the CFMs (Fig. S31). The method was found to be unsuitable for the quantification of gibberellin (GA) in TSB medium during the spiking experiment, likely due to matrix inferences or analyte instability; hence, GA was excluded from further analysis.

**Fig. 3:**
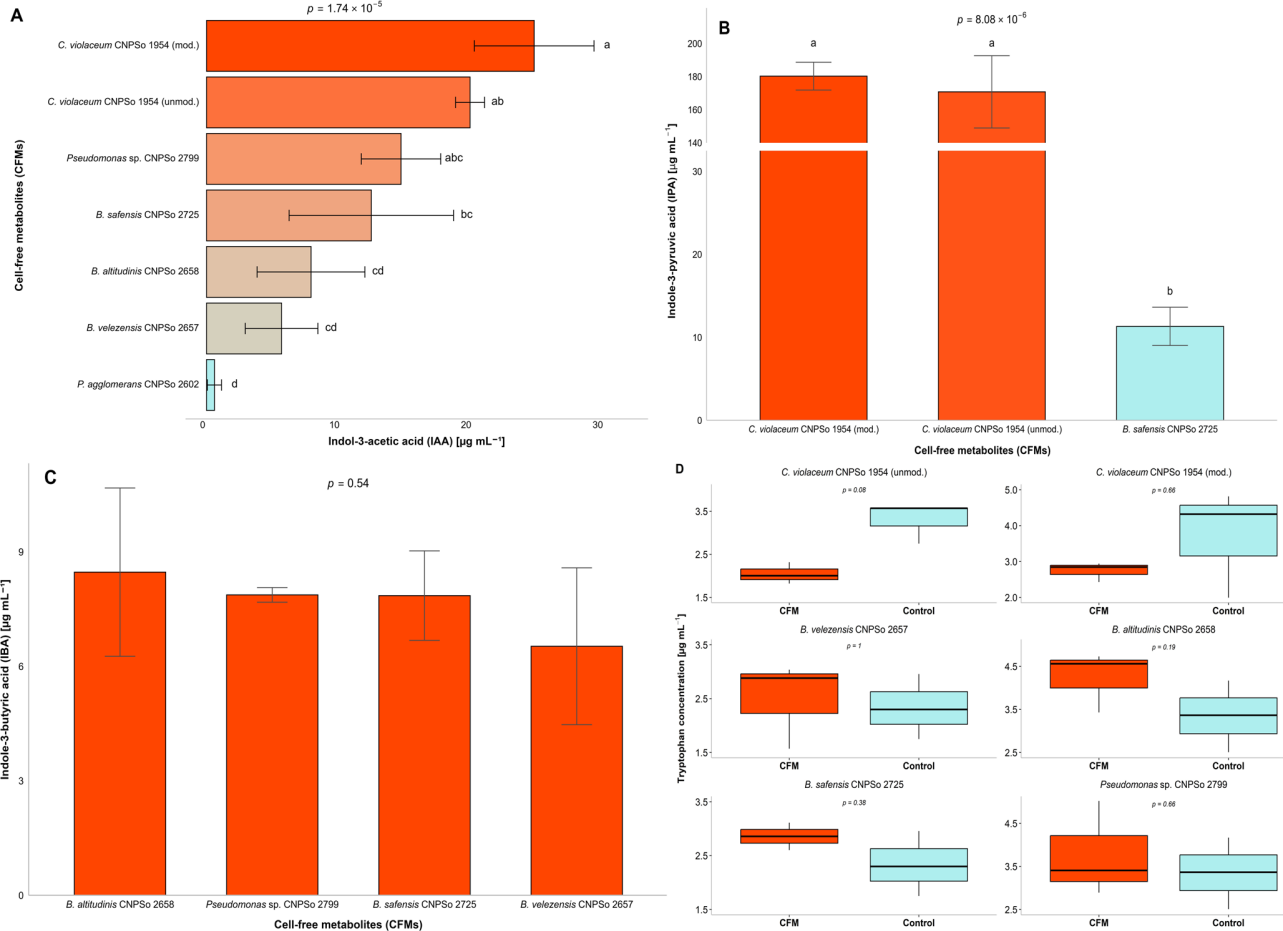
Phytohormone profiling in cell-free metabolites (CFMs) of six potential plants-growth promoting bacterial (PGPB) strains by RP-UPLC-PDA.** (A)** Quantitative detection of indole-3-acetic acid (IAA) (µg mL⁻¹) (*p* = 1.74 × 10^− 5^) produced by the strains CNPSo 1954, CNPSo 2602, CNPSo 2657, CNPSo 2658, CNPSo 2725, and CNPSo 2799, **(B)** Detection of indole-3-pyruvic acid (IPA) (µg mL⁻¹) (*p* = 8.08 × 10^− 6^), **(C)** Detection of indole-3-butyric acid (IBA) (µg mL^− 1^) (*p* = 0.54). Analyzed using Tukey’s test (*p* ≤ 0.05).** (D)** Quantitative detection of L-tryptophan (TRP) (µg mL⁻¹), where each CFMs sample from the strains CNPSo 1954 (unmod.) (*p* = 0.08), CNPSo 1954 (mod.) (*p* = 0.66), CNPSo 2657 (*p* = 1.00), CNPSo 2658 (*p* = 0.19), CNPSo 2725 (*p* = 0.38), and CNPSo 2799 (*p* = 0.66) were compared to its respective control using the Wilcoxon test (*p* ≤ 0.05). Each column represents a bacterial strain, analyzed in three biological replicates. Standard deviations (SD) are represented by error bars and different letters above the bars indicate significant differences between treatments (*n* = 3)

Indole-3-acetid acid (IAA) was the only phytohormone identified in all samples (Fig. 3A, Table S11). The CFMs of *C. violaceum* CNPSo 1954 exhibited the highest IAA concentrations, with 24.87 µg mL⁻¹ in the modified TSB medium, while in the other strains the concentrations, ranged from 0.60 to 14.76 µg mL⁻¹ in *P. agglomerans* CNPSo 2602 and *Pseudomonas* sp. CNPSo 2799, respectively (Fig. 3A, Table S11). The CFMs of *C. violaceum* CNPSo 1954 were also high in indole-3-pyruvic acid (IPA) in both TSB media, with an average of 175.55 µg mL⁻¹, while the lowest was detected in *B. safensis* CNPSo 2725, 11.32 µg mL⁻¹, being absent in the other strains (Fig. 3B, Table S11).

Indole-3-butyric acid (IBA) was detected in the CFMs of the three *Bacillus* strains and in *Pseudomonas* sp. CNPSo 2799, with concentrations ranging from 6.53 to 8.47 µg mL⁻¹, not differing significantly from each other (Fig. 3C, Table S11). This phytohormone was not detected in the CFMs of *C. violaceum* CNPSo 1954 (in both culture media) or in *P. agglomerans* CNPSo 2602 (Fig. 3C, Table S11). Abscisic acid (ABA) was detected only in the CFMs of *B. velezensis* CNPSo 2657, with 1.44 µg mL⁻¹, while trans-cinnamic acid (*t*-CA) was detected only in *C. violaceum* CNPSo 1954, 2.05 µg mL⁻¹ in TSB medium.

The L-tryptophan (TRP) levels in the controls ranged from 2.33 to 16.03 µg mL⁻¹, with the lowest values observed in *B. velezensis* CNPSo 2657 and *B. safensis* CNPSo 2725, and the highest for *P. agglomerans* CNPSo 2602 (Fig. 3D, Table S11). TRP was detected in all strain, except for *P. agglomerans* CNPSo 2602, ranging from 2.05 to 4.24 µg mL⁻¹ in *C. violaceum* CNPSo 1954 grown in unmodified medium and in *B. altitudinis* CNPSo 2658, respectively (Fig. 3D, Table S11). Overall, no statistically significant differences in TRP levels were observed between the treatments and their respective controls (Fig. 3D).

## Discussion

Beneficial microorganisms, as integral components of the microbial ecosystem, have been successfully utilized in a variety of bio-inputs, including inoculants biofertilizers, bio-stimulants, and biocontrol agents, enhancing plant growth, improving soil fertility, and mitigating environmental stresses (Liu et al. 2022; Li et al. 2023). Advances in biotechnology have allowed deeper insights into their roles, facilitating strategic deliveries in soil-plant systems (Jansson et al. 2023).

In a previous bioprospecting study by our group, we identified six strains that exhibited promising performance, including hydrolytic and proteolytic activities, tolerance to drought and high temperature, in maize grown under greenhouse conditions (Vasques et al. 2025). Aimed at their future use in agriculture, we sequenced their genomes, to obtain a proper taxonomic classification and get a deeper knowledge about their genes. Five strains had their taxonomic position defined; however, *Pseudomonas* sp. CNPSo 2799 was pointed out as a putative new species. In comparison to the closest type strain *Pseudomonas brassicacearum*, CNPSo 2799 showed an ANI value of 80.78% and a dDDH of 24.60%, well below the literature threshold values of 95–96% and 70%, respectively (Goris et al. 2007; Chun et al. 2018; Barco et al. 2020; Helene et al. 2022).

Natural competence is the bacterial ability to capture extracellular DNA. Once imported, the DNA can be metabolized to provide additional nucleotides or internalized into the chromosome and confer new genetic elements to the genome of the competent cell (Zuke et al. 2023). All *Bacillus* species analyzed in this study possess a set of competence genes (Table S7). This genetic repertoire can enhance uptake of exogenous DNA and promote chromosome remodeling and horizontal gene transfer (Burghard-Schrod et al. 2022; Denge et al. 2023). This DNA uptake capacity increases genome plasticity and contributes to the adaptation in diverse environments. Protein transport through the cell envelope is a process that contributes to cellular organization and integrity, constituting the basis of contact and exchange of substances between the cytosol and the external environment. The strains *C. violaceum* CNPSo 1954, *P. agglomerans* CNPSo 2602, and *Pseudomonas* sp. CNPSo 2799 presented different profiles of T1SS to T6SS. The nanomachines actively interact with the environment, hosts and other microorganisms. They can modulate secreted toxins and enzymes and manipulate host cells, circumventing immunity and eliminating competitors (Gupta et al. 2024). The presence of the Tad/Flp system present in *Pseudomonas* sp. CNPSo 2799 may confer adhesion to surfaces, representing an additional ecological advantage by facilitating colonization on surfaces and persistence in different environmental niches.

A comprehensive genomic analysis was performed to evaluate plant growth-promoting and potential biocontrol traits in the strains. Genes related to key metabolic pathways, including nitrogen and tryptophan metabolism and siderophore biosynthesis, support plant-microbe interactions, nutrient uptake, and stress tolerance (Chandrasekaran and Paramasivan 2024; Patel et al. 2024). The identification of these pathways provides a basis for optimizing PGPB through targeted genetic approaches, and the analyzed strains showed distinct metabolic features associated with plant growth promotion.

The bacterium *C. violaceum* is recognized for its genetic and metabolic versatility, particularly as a producer of violacein, a metabolite synthesized from the precursor tryptophan (Fig. 2), with significant pharmacological and agricultural potential. Violacein exhibits antifungal, nematicide, and insecticidal activities, representing a promising candidate for biocontrol applications in agriculture (Barreto et al. 2008; Durán et al. 2016). Indeed, *C. violaceum* CNPSo 1954 harbored genes related to siderophore production, phosphate solubilization, biofilm formation. The genome also encompasses the tryptophan biosynthesis pathway, consistent with the metabolomic profiling of phytohormones, which confirmed the strain’s ability to produce IAA and IPA, highlighting another important feature for potential applications in sustainable agriculture.

Certain species of *Pantoea* are well-known plant pathogens, for example, *P. agglomerans* pv. *gypsophilae* causes crown and root gall disease in gypsophila (*Gypsophila paniculata*), while *P. agglomerans* pv. *betae* infects beets (*Beta vulgaris* L) (Dutkiewicz et al. 2016a). However, *P. agglomerans* occupies a wide range of ecological niches, and may be an epiphyte, endophyte, or rhizospheric in plant interactions. This versatile bacterium is also described as being able to promote plant growth, including nitrogen fixation, phytohormone production, phosphate solubilization, enhancing stress tolerance, and providing biocontrol capabilities (Lorenzi et al. 2022; Dahiya et al. 2024). For instance, antimicrobials produced by *P. agglomerans* are used as biocontrol agents in commercial products to combat fire blight (*Erwinia amylovora*) in apple (*Malus domestica* B.) and pear (*Pyrus communis* L.) orchards (Johnson and Stockwell 1998; Llontop et al. 2020). Moreover, certain strains of *P. agglomerans* have been shown to confer systemic acquired resistance in some plant species (Soluch et al. 2021). The distinction between pathogenic and non-pathogenic species and strain based solely on their genomes remains challenging. *P. agglomerans*, as a facultative anaerobic bacterium, demonstrates markedly different behaviors under varying environmental conditions, switching between distinct metabolic pathways (Rezzonico et al. 2009; Patakova et al. 2024). *P. agglomerans* CNPSo 2602, evaluated in this study, represents an example of an endophytic bacterium within the genus, confirming genes associated with nitrogen metabolism, biofilm formation, siderophore production, and phosphate solubilization. In the study by Vasques et al. (2025), strain CNPSo 2602 exhibited phosphorus solubilization in *in vitro* assays; additionally, it possesses the tryptophan biosynthesis pathway, and metabolomic analysis confirmed its ability to produce IAA.

The genus *Bacillus* comprises environmentally versatile species capable of inhabiting diverse and extreme conditions, largely due to their ability to form stress-resistant endospores (Kolomiiets et al. 2024; Valencia-Marin et al. 2024). *Bacillus* strains find extensive applications in agriculture, both in biocontrol and as biofertilizers due to their genomic machinery, with the synthesis of a variety of secondary metabolites with antimicrobial activity and molecules that can stimulate plant growth (Fira et al. 2018; Valencia-Marin et al. 2024; Vasques et al. 2024). Among the *Bacillus* species, *B. velezensis*,* B. altitudinis*, and *B. safensis* are recognized for antimicrobial activity, pathogen suppression, stresses tolerance induction, and plant growth promotion (Lateef et al. 2015; Rabbee et al. 2023; Zhang et al. 2023). Consistently, the three *Bacillus* strains analyzed in this study harbored genes related to antibiotic and siderophore biosynthesis and the tryptophan biosynthetic pathway. Metabolomic analysis confirmed the synthesis of phytohormones such as IAA and IBA.

Bacteria of the genus *Pseudomonas* compose one of the most extensively studied groups of microorganisms for the biocontrol of bacterial and fungal diseases. Several species, including *P. fluorescens*,* P. putida*,* P. chlororaphis* and *P. koreensis*, have demonstrated strong biocontrol potential, primarily due to their intrinsic ability to synthesize metabolites with antifungal and antibacterial activities (Kumar et al. 2015, 2025; Goswami et al. 2016; Kolomiiets et al. 2024; Plokhovska et al. 2025). Beyond phytopathogen suppression, these bacteria can enhance plant biomass production by facilitating nutrient acquisition (Ramakrishna et al. 2019), support plant resilience under drought (Kalleku et al. 2024; Kálmán et al. 2024), salt stresses (Hernández-Canseco et al. 2022; Maurya et al. 2024), and contribute to the biodegradation of contaminated soils (Ma et al. 2017; Chandrasekaran and Paramasivan 2024). *Pseudomonas* sp. CNPSo 2799 harbors genes related to nitrogen metabolism, biofilm formation, and the tryptophan biosynthesis pathway. Metabolomic profiling further confirmed the ability to synthetize IAA and IBA, reinforcing its potential as a sustainable bioinoculant for agriculture. In the study by Vasques et al. (2025), strain CNPSo 2799 exhibited biofilm formation, IAA production, siderophore synthesis, and exopolysaccharide production *in vitro* assays. Nevertheless, it is important to note that certain *Pseudomonas* species are opportunistic pathogens, capable of causing diseases in humans, animals, and plants (Holloway 1969; Fernandes et al. 2009; Lamichhane et al. 2014).

All six strains exhibited a significant portion of their genomes dedicated to the secondary metabolites BGCs with broad-spectrum antibacterial and antifungal properties. These findings are consistent with previous studies, e.g., Fira and collaborators (2018) reported that approximately 5–8% of the genomes of species belonging to the *Bacillus* genus are dedicated to the secondary metabolites BGCs, including antagonistic compounds. Furthermore, for three strains (LGMB12, LGMB319, and LGMB426) of *B. velezensis*, Ercole and collaborators (2024) reported that an average of 16.2% of the genomes of this species are dedicated to secondary metabolite BGCs. These findings are consistent with our results, as *B. velezensis* CNPSo 2657 exhibited 18.03% of its genome dedicated to secondary metabolite BGCs, highlighting its potential for multiple applications in sustainable agriculture. While *B. altitudinis* CNPSo 2658 and *B. safensis* CNPSo 2725 showed an average of 8.29%.

We identified CAZymes involved in polysaccharide hydrolysis (Levy et al. 2018; Tashkandi et al. 2022). Their enrichment in these microbiomes is believed to result from the reciprocal interaction between plant exudates and microbial enzymatic activity, promoting synergistic benefits between plants and microbes (Sonbol and Jalal 2025). The CAZymes analysis of all six strains are linked to fungal cell wall degradation, biofilm production and microbial colonization (Tsalgatidou et al. 2022). Understanding the regulatory mechanisms of these enzymes represents a critical step to enhance microbial capabilities for sustainable agricultural applications (Wash et al. 2023).

However, it is worth mentioning that PGPB strains may also harbor antibiotic resistance genes (ARGs) and virulence factors that could pose potential risks to human health. Therefore, it is essential to understand the relationship between the presence of ARGs in bacterial genomes and their potential transfer to other bacteria in the rhizosphere through horizontal gene transfer, which may promote the dissemination of antimicrobial resistance across different microbial communities. It is essential to assess these biosafety concerns, addressing public health implications and establishing a regulatory framework that includes practical solutions and recommendations. Therefore, it is mandatory to adopt rigorous approaches for the isolation, characterization, and development of effective and biosafe PGPB-based biofertilizers (Mahdi et al. 2022). To verify if the strains carry both putative pathogenic and plant growth-promoting properties, we bioprospected their genomes. Regarding antibiotic resistance genes, *B. velezensis* CNPSo 2657 exhibited resistance to only three antibiotics, *B. altitudinis* CNPSo 2658 and *B. safensis* CNPSo 2725 to four, and *C. violaceum* CNPSo 1954 and *Pseudomonas* sp. CNPSo 2799 to five antibiotics. Notably, *P. agglomerans* CNPSo 2602 raised a potential biosafety concern, exhibiting resistance to 12 different antibiotics, comparable to that observed in the KM1 strain, whose genome harbors 13 ARGs conferring resistance to clinically relevant drugs such as penicillin G, bacitracin, rifampin, vancomycin, and fosfomycin (Guevarra et al. 2021). However, the antibiotics to which strain CNPSo 2602 exhibits resistance are not commonly used in human or veterinary medicine, according to the WHO Bacterial Priority Pathogens List (2024), such as spectinomycin, which is naturally synthesized by other bacterial species. Nevertheless, understanding antibiotic resistance in agricultural systems, particularly those involving bacteria used in biofertilizer application, is essential to assess the potential for selection of ARGs that may pose risks to human health. Furthermore, if clinically relevant ARGs are detected in biofertilizers, it becomes imperative to investigate whether their frequency or host range is altered upon exposure to specific microbial strains (Mahdi et al. 2022). Although CNPSo 2602 strain did not exhibit genes associated with horizontal gene transfer in our analysis, either for acquisition or transfer, its use as a biofertilizer cannot be recommended without prior rigorous risk assessment, including biosafety evaluations to ensure its applicability without posing risks to the environment, animals, or human health. The bacterium *P. agglomerans* possesses numerous beneficial traits that can be exploited for the prevention and/or treatment of human and animal diseases, the control of plant pathogens, the promotion of plant growth, and environmental bioremediation. Despite the documented pathogenic potential of *P. agglomerans* in causing occupational diseases of allergic and/or immunotoxin origin and occasional infections, the beneficial characteristics of these species, as well as those of related species within the genus *Pantoea*, remain of considerable value for potential applications across multiple areas of biotechnology (Dutkiewicz et al. 2016b).

We also conducted a targeted metabolic analysis of the CFMs, focusing on phytohormones and associated compounds, key molecules regulating plant growth and stress responses (Sabagh et al. 2022). Among them, IAA should be emphasized, as an important promoter of rhizosphere colonization and root development (Orozco-Mosqueda et al. 2023). IAA may also facilitate nutrient solubilization activity in IAA-synthesis bacteria (Alemneh et al. 2021) and enhance plant tolerance to drought, metal stress, and salinity (Etesami and Glick 2024). IAA was identified in the CFMs of all six strains, with *C. violaceum* CNPSo 1954 showing the highest synthesis in both TSB and modified-TSB culture media. Similarly, Vasques et al. (2025) also highlighted this strain as the greatest IAA production among 100 PGPB strains using the Salkowski assay (Gordon and Weber 1951; Sarwar and Kremer 1995).

The specific mode of IAA synthesis by PGPB is rarely elucidated due to the complexity of IAA biosynthesis and regulation, the promiscuity of enzymes involved in its biosynthetic pathways, and the existence of three distinct pathways classified based on their intermediates: IAN, IAM, and IPA. Some enzymes involved in IAA biosynthesis are known to be regulated by IAA itself or its precursors, as well as by a wide range of environmental signals (Duca and Glick 2020). Consequently, multiple genes are involved in IAA biosynthesis, which can vary among organisms, ultimately leading to IAA synthesis. Our *in* *silico *findings (Fig. 2) are consistent with metabolomic analyses, which revealed that all strains possess genes associated with pathways involved in IAA biosynthesis. *P. agglomerans* strain CNPSo 2602 exhibited the lowest diversity and concentration of phytohormonal compounds among all strains analyzed, synthesizing only IAA. Genomic predictions for this strain revealed the presence of genes, such as *iaaH*, *iad*, *aldH*, and *ipdC* associated with tryptophan metabolism and IAA biosynthesis. However, the metabolomic data showed limited IAA production and complete TRP depletion in the CFMs, suggesting a divergence between genomic potential and metabolic output. This discrepancy may reflect transcriptional regulation, metabolic channeling of TRP into alternative pathways, or condition-dependent gene expression. Future studies employing transcriptomic or fluxomic approaches will be essential to validate the activity of these predicted pathways and to clarify the metabolic fate of TRP in this strain. Notably, TRP was not detected in the CFMs of this strain, despite its presence in the respective control medium, which contained mannitol and yeast extract, and exhibited the highest qualified levels. This absence may suggest active TRP consumption by the strain. However, even with consumption, the strain synthesized only a small amount of IAA in comparison to the other strains, indicating that the precursor may have been diverted to alternative metabolic pathways. Supporting this hypothesis, previous studies have shown that IAA biosynthesis can proceed via multiple tryptophan-dependent routes, including IAM, ILA, IAN, and IAAld, often leading to further conversion into other indole derivatives such as IPA and IBA (Szkop and Bielawski 2013; Goswami et al. 2015).

Interestingly, despite the low IAA synthesis observed in our study, Vasques et al. (2025) reported a high IAA concentration for the same strain cultivated in modified-YM medium supplemented with L-tryptophan and incubated for seven days. Similarly, Luziatelli et al. (2020a) reported that the *P. agglomerans* strain C1 synthetized IAA when grown in LB medium supplemented with L-tryptophan. Further investigations by the same group (Luziatelli et al. 2020b) highlight that not only TRP, but also the medium’s carbon source and yeast extract, significantly influence the exometabolome, including IAA synthesis. Sucrose-enriched medium promoted higher IAA levels, reinforcing the need to consider both nutritional and environmental factors in optimizing phytohormone biosynthesis. Bioprospecting of novel microorganisms is of paramount importance, as strains belonging to the same species may exhibit unique characteristics regarding enzyme activity, nutrient accumulation, natural life cycle, and growth habits, all of which contribute to their distinct growth-promoting capabilities (Ercole et al. 2025).

For *C. violaceum* CNPSo 1954, cultivation conditions appeared to play a critical role in phytohormone synthesis. This strain was grown at 200 rpm, while the others were cultured at 100 rpm, a difference that may have contributed to its markedly higher IAA levels in modified medium, nearly twice the highest values observed in the other strains. A higher rotation speed was employed in *C. violaceum* CNPSo 1954 because, in previous experiments conducted by our group, we observed that violacein production can be inhibited at lower agitation rates due to the effect of glucose on dissolved oxygen levels, which affects the conversion of tryptophan into this pigment (DeMoss and Evans 1959; Ahmad et al. 2012). Despite using the same incubation parameters, *C. violaceum* CNPSo 1954 cultivated in unmodified medium showed performance that did not differ statistically from that in the modified medium, yet did not differ from other strains exhibiting lower IAA production, emphasizing the importance of medium composition. According to Duca and Glick (2020), IAA biosynthesis is regulated by complex enzymatic systems influenced by factors such as pH, temperature, and nutrient availability. Moreover, although TRP was detected in both the CFMs and in the culture medium used for CNPSo 1954 cultivation, no significant statistical difference was observed between them, suggesting that the detected TRP originated primarily from the medium rather than *de novo* synthesis or release by the bacterium. These findings support the hypothesis that optimal conditions for microbial growth do not necessarily coincide with optimal conditions for IAA synthesis. Previous research has reported *C. violaceum* as a producer of indole compounds, including IAA (Corpe 1961; Durán et al. 1994; Hoshino 2011), yet the metabolic flexibility and regulation of this biosynthetic process remain poorly understood. *C. violaceum* CNPSo 1954 also showed high IPA synthesis in both culture media. IPA is a key intermediate in tryptophan-dependent IAA biosynthesis pathway, underscoring its pivotal role in phytohormone synthesis (Imada et al. 2017). Our results showed that this intermediate molecule was overproduced compared to IAA. Although metabolomic tests confirmed that only the bacterial strains *C. violaceum* CNPSo 1954 and *B. safensis* CNPSo 2725 synthetized IPA (Table 5, Table S11), since IPA is a precursor of IAA (Duca and Glick 2020), it may have been converted into IAA rather than being expressed as IPA. Nevertheless, the mere presence of a gene does not necessarily indicate its expression, requiring biochemical assays for validation in technological applications (Goeman and Bühlamann 2007; Ambrosini and Passaglia 2018).

We also detected IBA in the CFMs of all three *Bacillus* strains and *Pseudomonas* sp. CNPSo 2799. A review by Frick and Strader (2018) highlighted IBA’s role as a precursor of IAA in plant development. This conversion pathway plays a distinct role in modulating auxin levels during plant growth, particularly under stress, in ways not compensated by other pathways. Therefore, a single biosynthetic pathway can produce multiple phytohormones, with individual genes participating in several pathways (Duca and Glick 2020). *B. velezensis* CNPSo 2657 was the only strain able to synthesize ABA, a phytohormone usually produced in plants via the carotenoid pathway. ABA regulates the balance between growth and defense, accumulating under stress and returning to basal levels under normal conditions. Its activity involves antagonism with gibberellins and melatonin, synergy with auxins and salicylic acid, and context-depending on interactions with ethylene, influencing seed dormancy, root growth, and stress responses (Chen et al. 2020; Singh and Roychoudhury 2023). Additionally, only *C. violaceum* CNPSo 1954 produced *t-*CA, an important intermediate in plant secondary metabolism via the phenylpropanoid pathway by the action of phenylalanine ammonia-lyase (PAL) (Hoskins 1984). Its synthesis was observed exclusively in unmodified TSB medium, likely due to the presence of glucose, which is known to initiate the phenylpropanoid pathway and stimulate lignin biosynthesis (Wang et al. 2024). Despite being found in trace amounts in plants, *t-*CA exhibits allelopathic activity, influencing plant growth either positively or negatively (Lupini et al. 2016).

## Conclusions

The genomic analyses of *C. violaceum* CNPSo 1954, *P. agglomerans* CNPSo 2602, *B. velezensis* 2657, *B. altitudinis* CNPSo 2658, *B. safensis* CNPSo 2725, and of the putative novel specie *Pseudomonas* sp. CNPSo 2799 reveal significant potential as plant growth-promoting and biocontrol agents. Secondary metabolites BGCs, particularly in *B. velezensis* CNPSo 2657 (18.03% of the genome), alongside with CAZymes, with particular emphasis on the glycoside hydrolase and glycosyltransferase enzyme classes, representing more than 50.7% of the CAZymes repertoire across the six strains, underscores their biocontrol activity. Additionally, these strains have demonstrated the ability to synthesize key phytohormones, especially IAA, which was produced by all strains and play crucial roles in plant development and stress responses. However, qualitative and quantitative variation among the strains highlights the importance of bioprospecting at the strain level to identify those with the greatest contribution. Overall, our findings support the potential of these strains as bio-inputs, in a new category of multifunctional microorganisms, combining plant growth-promoting with biocontrol activities, thereby contributing to sustainable agricultural practices.

## Supplementary Information

Below is the link to the electronic supplementary material.

Supplementary Material 1.

Supplementary Material 2.

## Data Availability

All data are presented or indicated in the main manuscript or as supplementary material.
