# Supplementary material for: Integrated genome mining and phytohormone profiling of six plant growth-promoting elite bacterial strains
Source: Arch Microbiol. 2026 Jan 21;208(3):152. doi: 10.1007/s00203-025-04712-6 (PMC12823646; doi:10.1007/s00203-025-04712-6)
Supplement: Supplementary file 2 — Supplementary Material 2. [file 203_2025_4712_MOESM2_ESM.docx]

**Integrated genome mining and phytohormone profiling of six plant growth-promoting elite bacterial strains**

Tairine Graziella Ercole, Rafaella Liviero, Leonardo Araujo Terra, Guilherme Julião Zocolo, Milena Serenato Klepa, Renan Augusto Ribeiro, Marco Antonio Nogueira, Mariangela Hungria

**SUPPLEMENTARY FIGURES**


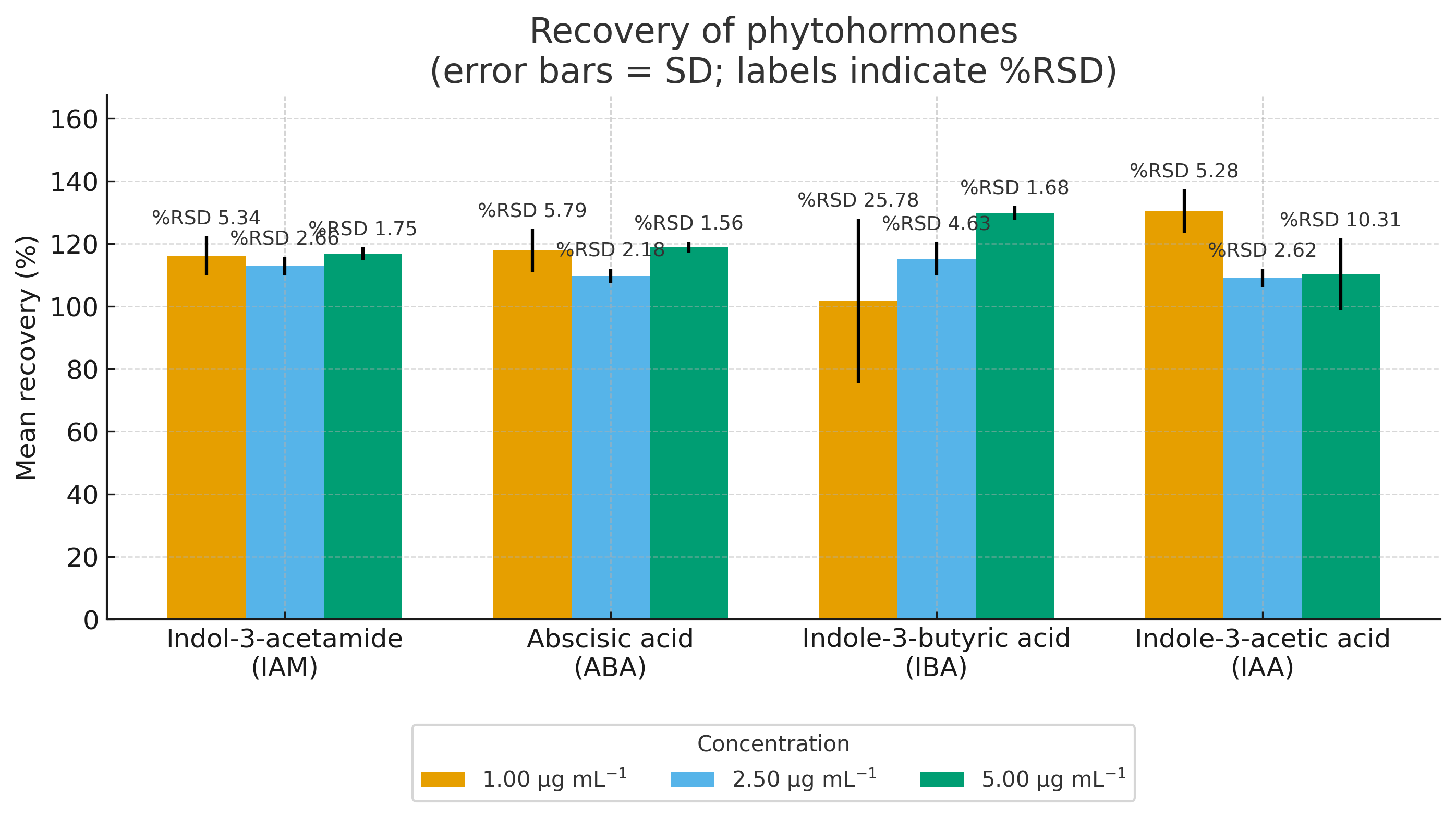


**Fig. S1.** Mean recovery (%) and standard deviation for phytohormones at three fortification levels (1.0, 2.5 and 5.00 µg mL⁻¹) in DYGS medium. Both compounds were selected as representative phytohormones to support method validation.


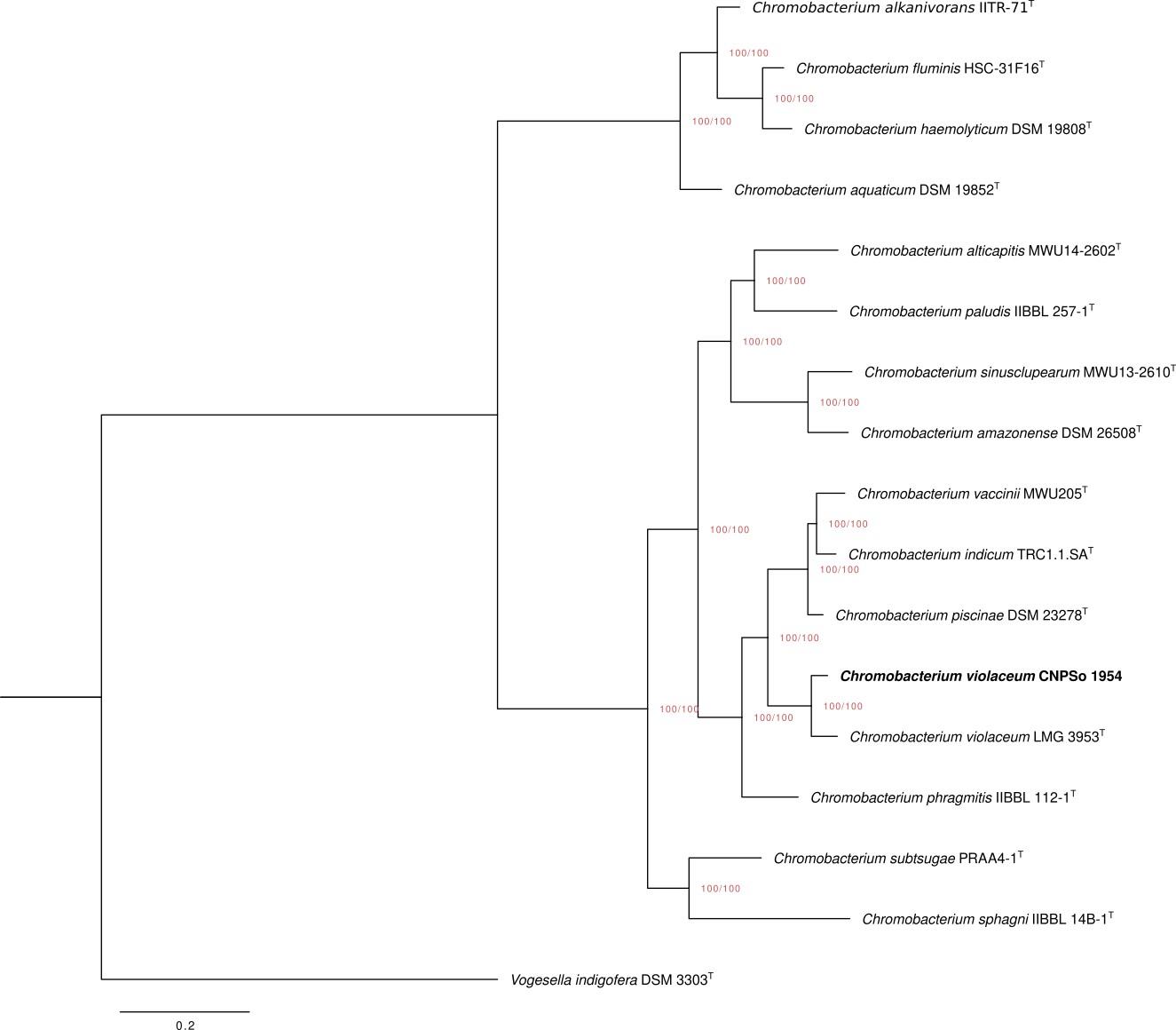


**Fig. S2.** Phylogenetic analysis of species within the genus *Chromobacterium* based on 381 conserved universal marker genes by PhyloPhlAn 3.0, reconstructed with the LG+F+R2 replacement model. The target strain is highlighted in bold.


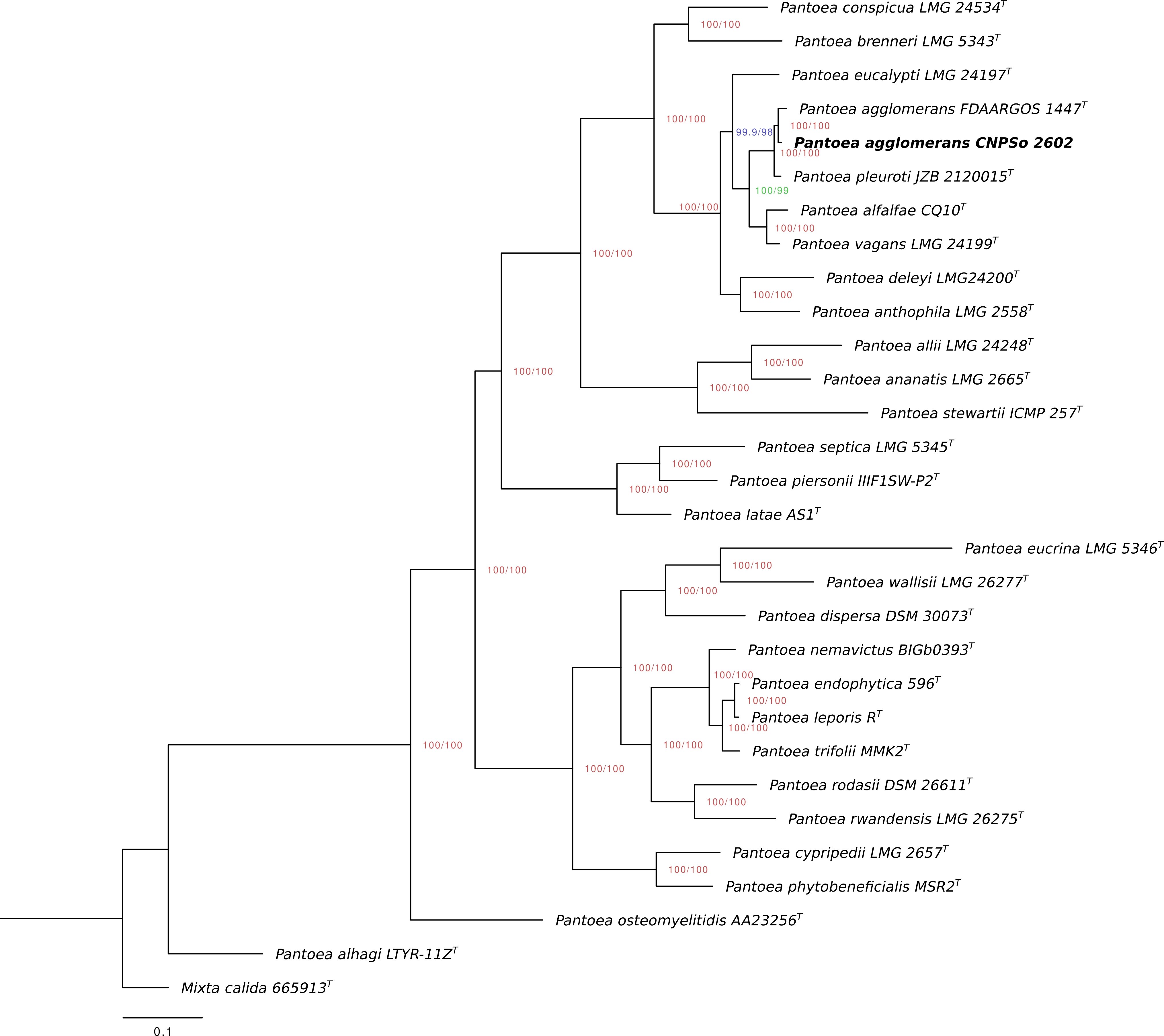


**Fig. S3.** Phylogenetic analysis of species within the genus *Pantoea* based on 374 conserved universal marker genes identified by PhyloPhlAn 3.0, reconstructed with the WAG+F+R3 replacement model. The target strain is highlighted in bold.


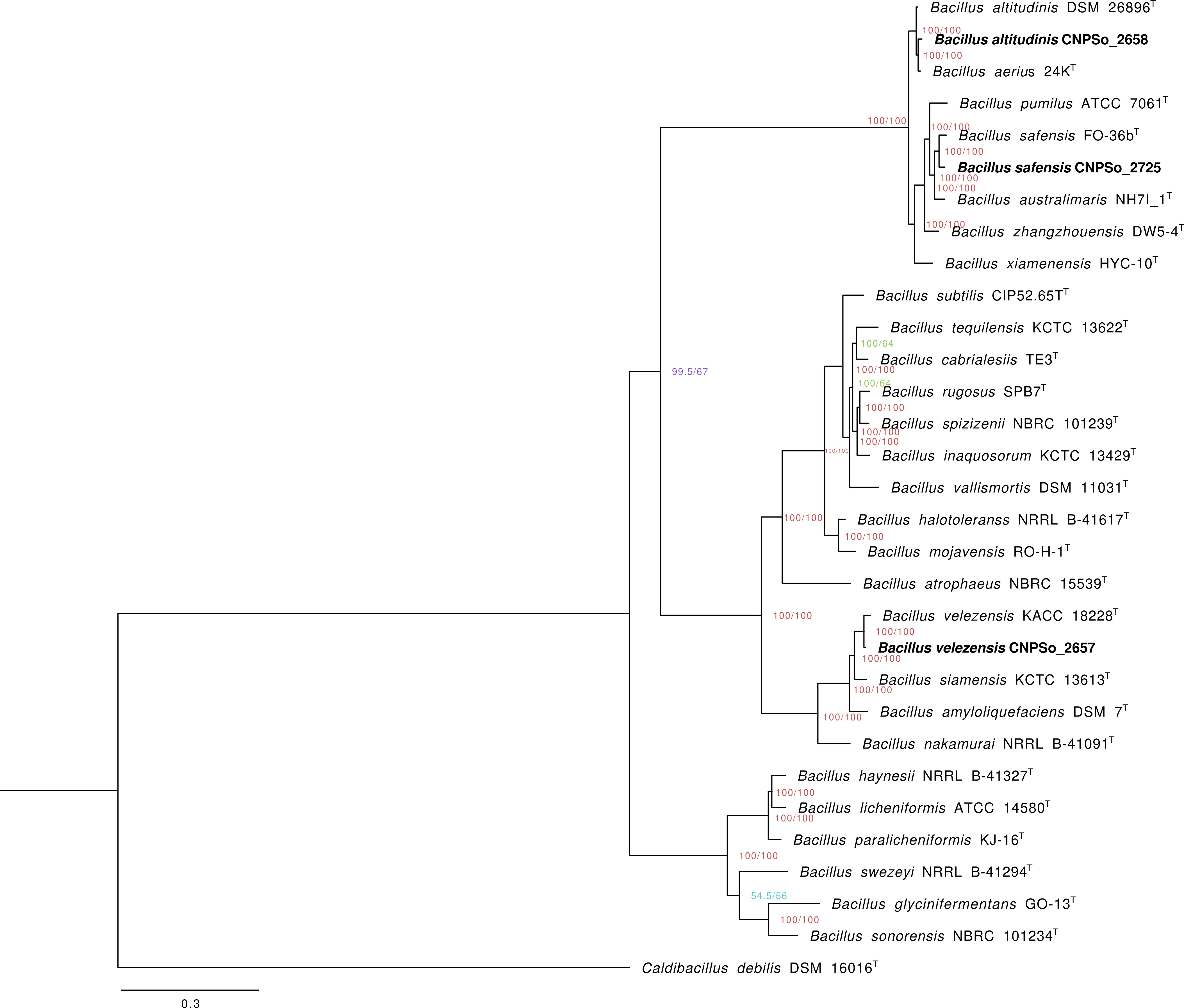


**Fig. S4.** Phylogenomic analysis of *Bacillus* species based on 384 conserved universal marker genes, identified by PhyloPhlAn 3.0 and reconstructed under the JTT+F+R2 replacement model. Only the phylogenetically closest *Bacillus* species were included. Target strains are highlighted in bold.


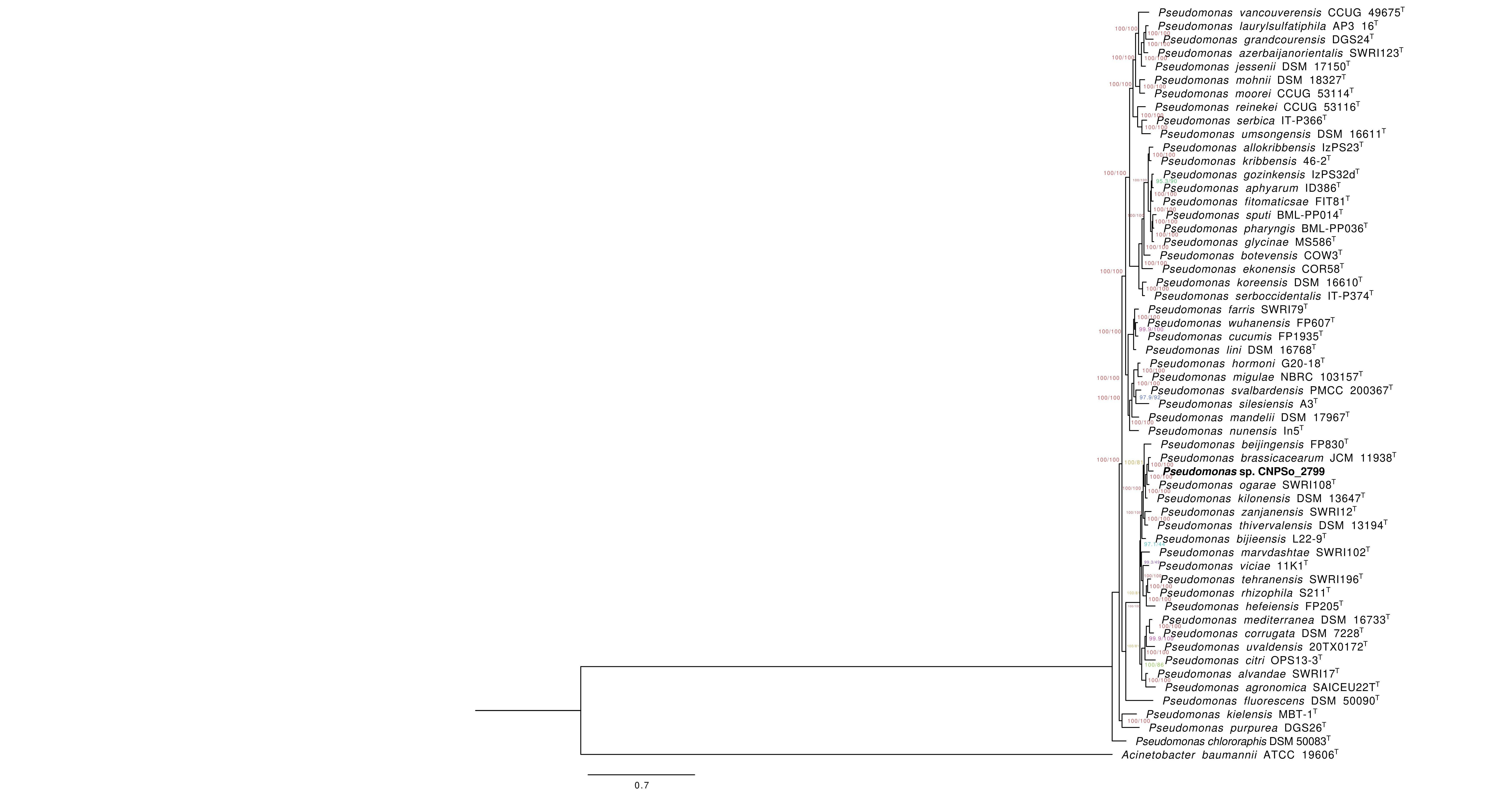


**Fig. S5.** Phylogenomic analysis of species of the genus *Pseudomonas* based on 387 conserved universal marker genes, identified by PhyloPhlAn 3.0 and reconstructed under the VT+F+R2 replacement model. Only the phylogenetically closest *Pseudomonas* species were included. The target strain is highlighted in bold.

**Fig. S6.** The protein-coding genes (PCGs) and their corresponding quantities in bacteria strains CNPSo 1945 (blue column), CNPSo 2602 (orange column), CNPSo 2657 (gray column), CNPSo 2658 (red column), CNPSo 2725 (green column), and CNPSo 2799 (black column) annotated from the complete genome utilizing the RAST and SEED Viewer online annotation server. The coverage of the protein subsystems detected was at least 72 %.


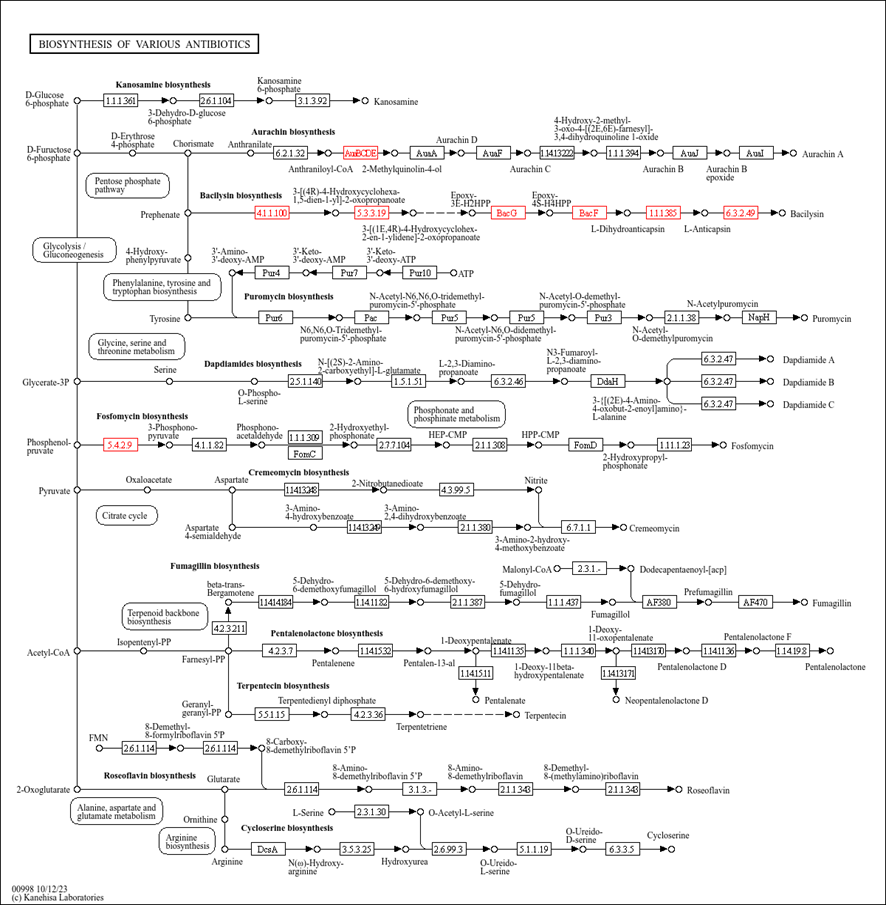


**Fig. S7.** Predicted antibiotic biosynthesis pathways of *Bacillus velezensis* CNPSo 2657, inferred from The Kyoto Encyclopedia of Genes and Genomes (KEGG) database (http://www.kegg.jp).


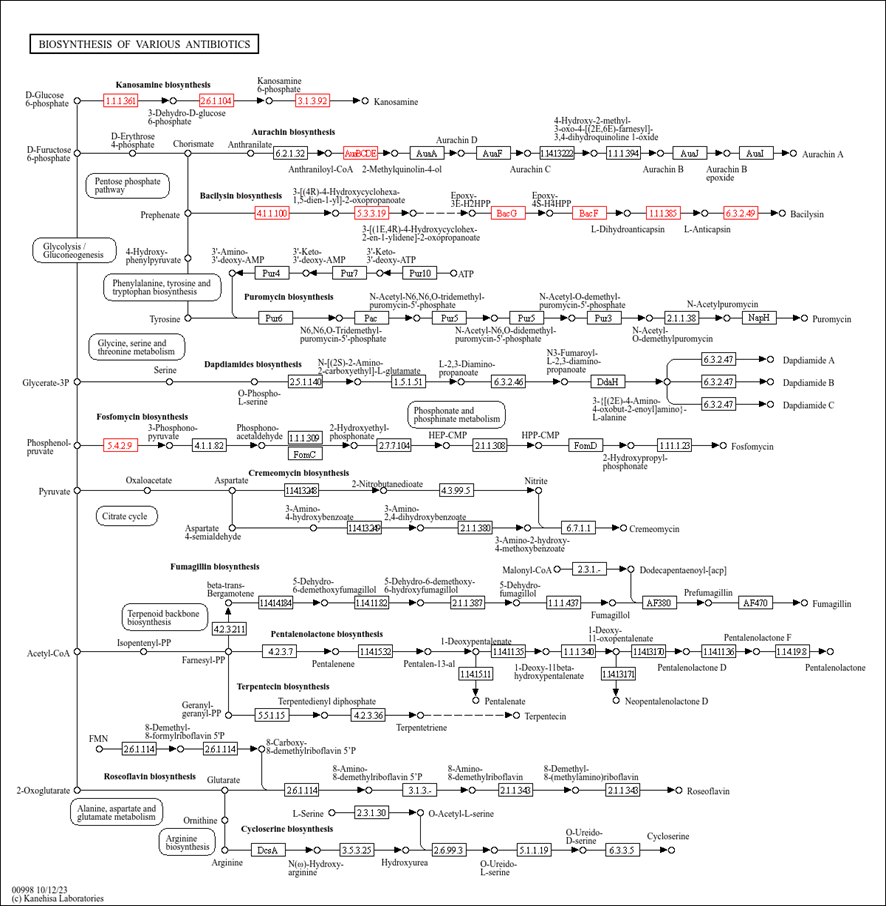


**Fig. S8.** Predicted antibiotic biosynthesis pathways of *Bacillus altitudinis* CNPSo 2658, inferred from The Kyoto Encyclopedia of Genes and Genomes (KEGG) database (http://www.kegg.jp).


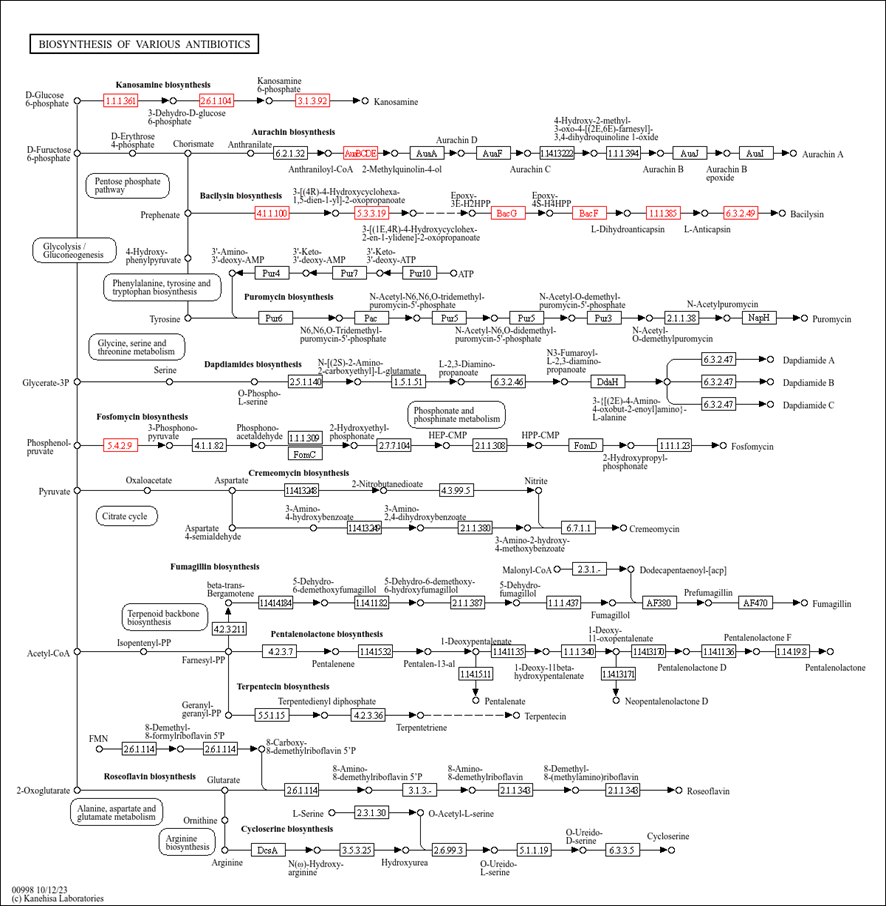


**Fig. S9.** Predicted antibiotic biosynthesis pathways of *Bacillus safensis* CNPSo 2725, inferred from The Kyoto Encyclopedia of Genes and Genomes (KEGG) database (http://www.kegg.jp).


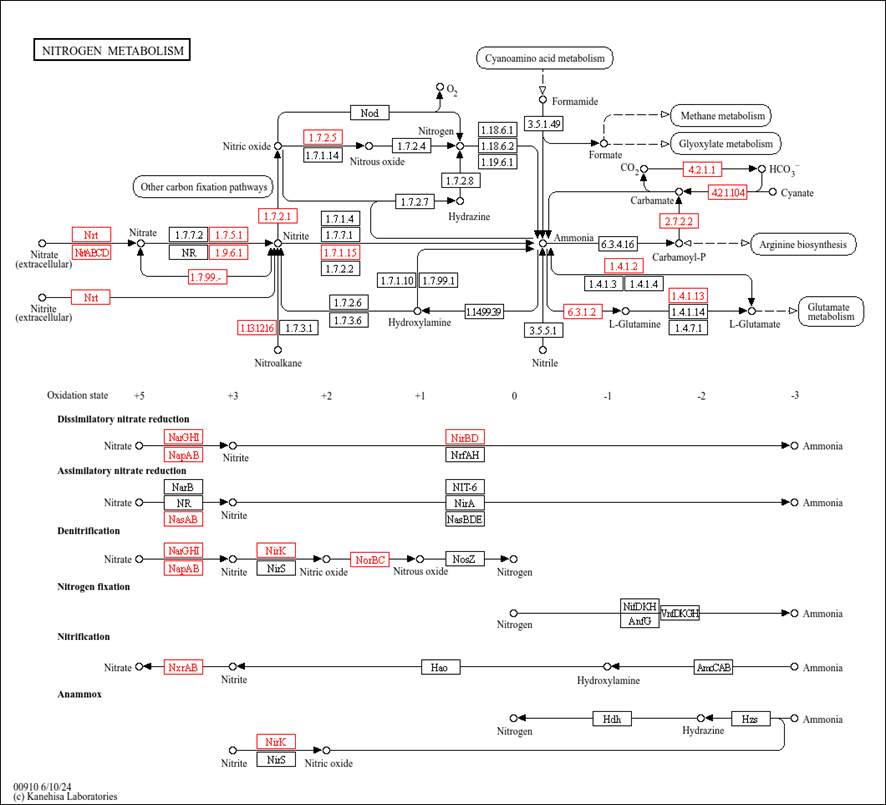


**Fig. S10.** Predicted nitrogen metabolism pathway of *Chromobacterium violaceum* CNPSo 1954 based on The Kyoto Encyclopedia of Genes and Genomes (KEGG) database (http://www.kegg.jp).


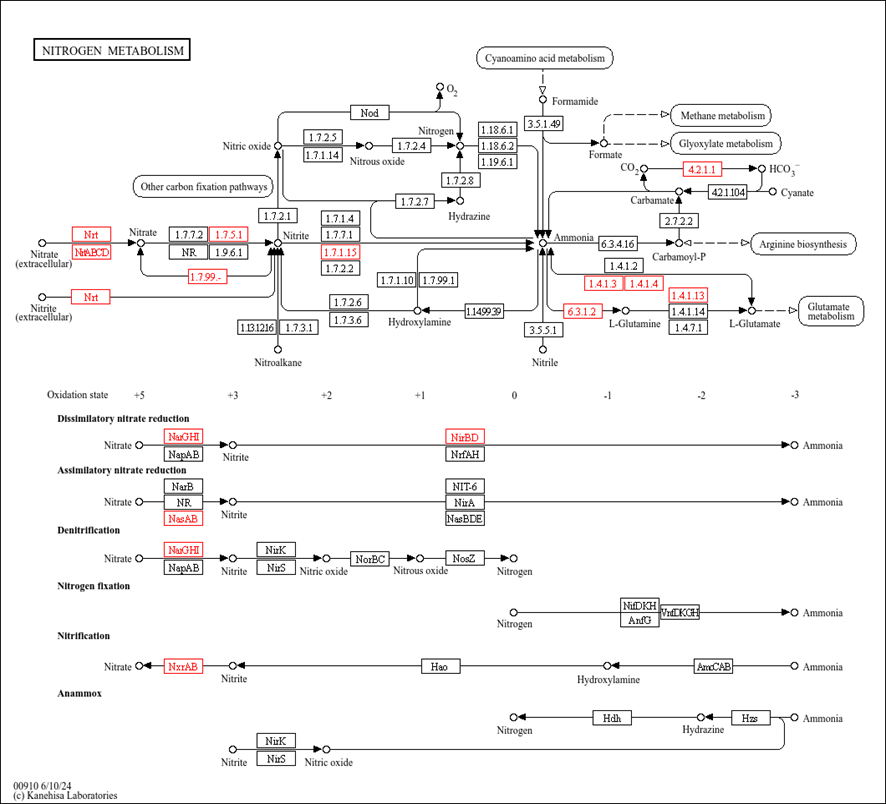


**Fig. S11.** Predicted nitrogen metabolism pathway of *Pantoea agglomerans* CNPSo 2602 based on The Kyoto Encyclopedia of Genes and Genomes (KEGG) database (http://www.kegg.jp).


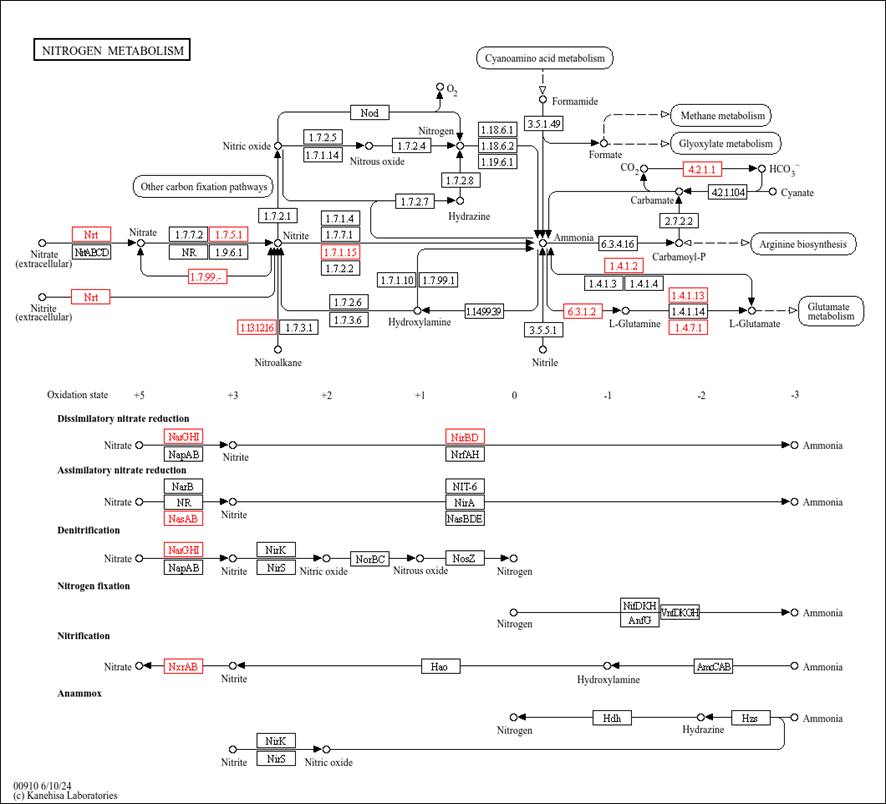


**Fig. S12.** Predicted nitrogen metabolism pathway of *Bacillus velezensis* CNPSo 2657 based on The Kyoto Encyclopedia of Genes and Genomes (KEGG) database (http://www.kegg.jp).


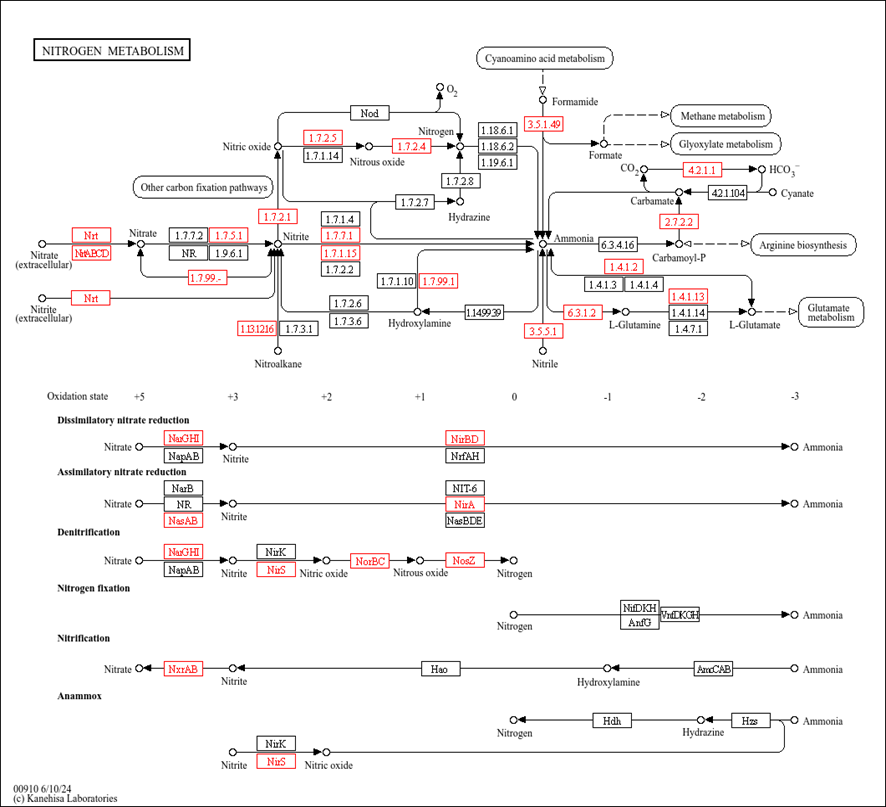


**Fig. S13.** Predicted nitrogen metabolism pathway of *Pseudomonas* sp. CNPSo 2799 based on The Kyoto Encyclopedia of Genes and Genomes (KEGG) database (http://www.kegg.jp).


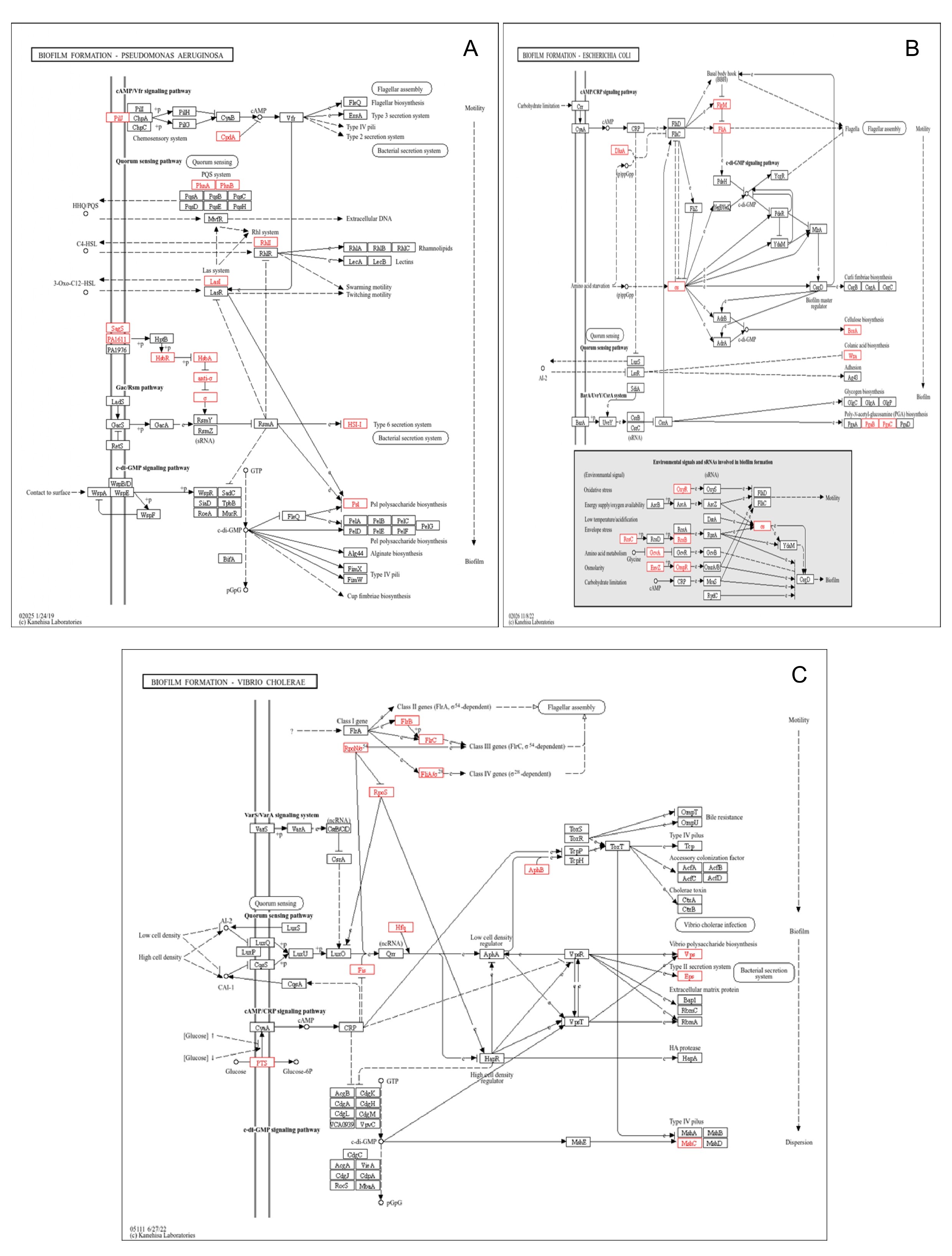


**Fig. S14.** Predicted biofilm formation pathways of *Chromobacterium violaceum* CNPSo 1954, inferred from The Kyoto Encyclopedia of Genes and Genomes (KEGG) database (http://www.kegg.jp). A) Based on *Pseudomonas aeruginosa* reference pathway; B) Based on *Escherichia coli* reference pathway; and C) Based on *Vibrio cholerae* reference pathway.


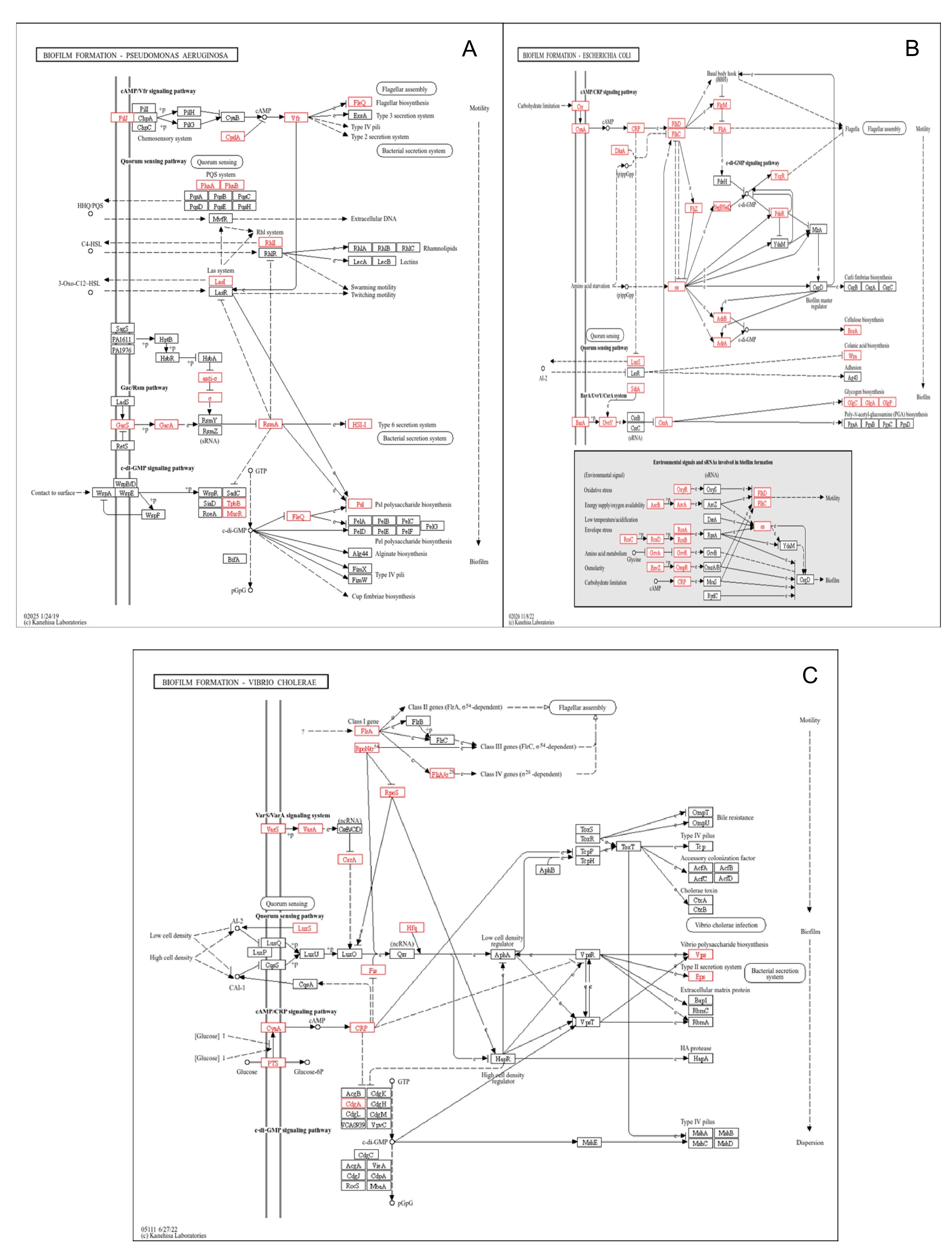


**Fig. S15.** Predicted biofilm formation pathways of *Pantoea agglomerans* CNPSo 2602, inferred from The Kyoto Encyclopedia of Genes and Genomes (KEGG) database (http://www.kegg.jp). A) Based on *Pseudomonas aeruginosa* reference pathway; B) Based on *Escherichia coli* reference pathway; and C) Based on *Vibrio cholerae* reference


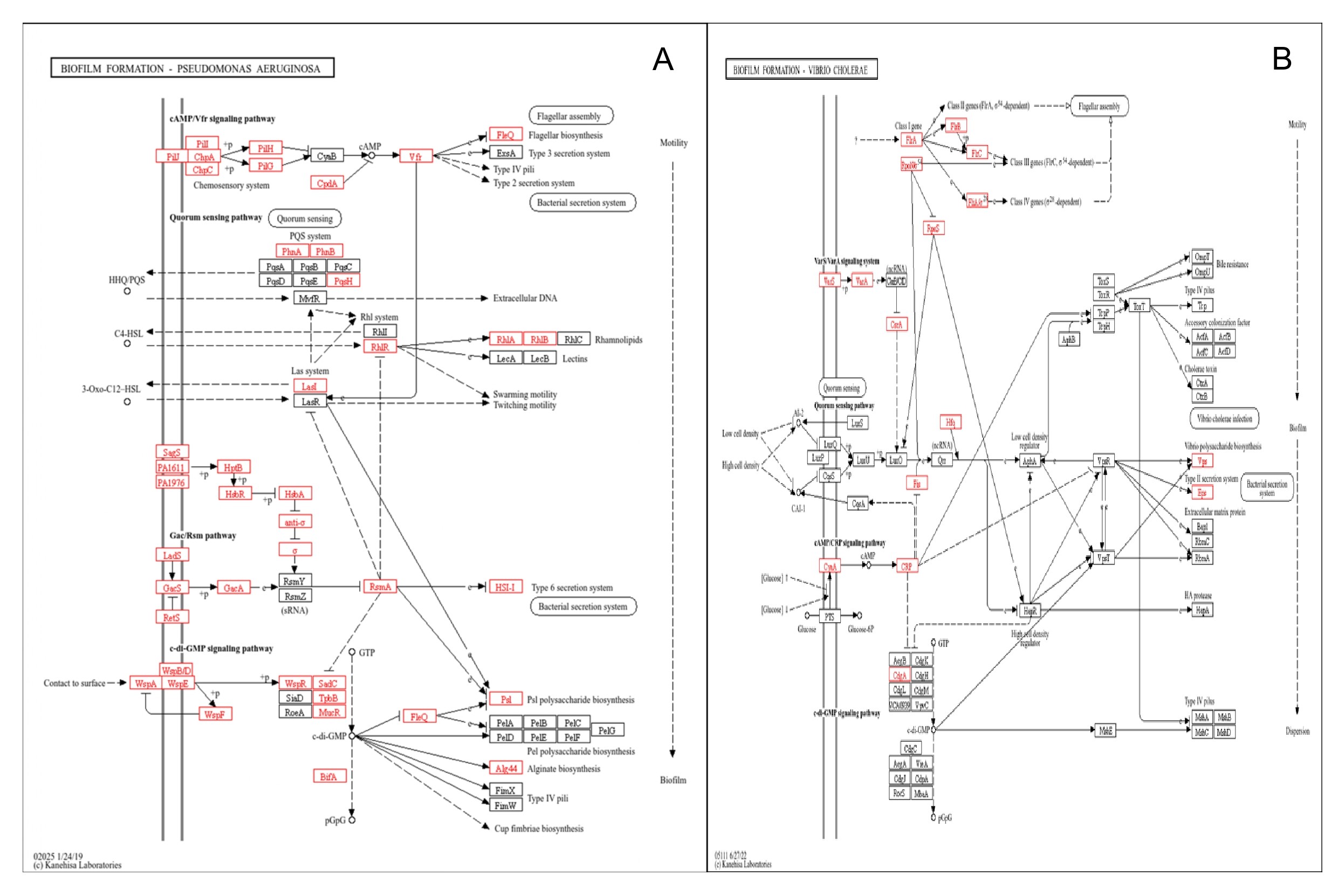


**Fig. S16.** Predicted biofilm formation pathways of *Pseudomonas* sp. CNPSo 2799, inferred from The Kyoto Encyclopedia of Genes and Genomes (KEGG) database (http://www.kegg.jp). A) Based on *Pseudomonas aeruginosa* reference pathway; and B) Based on *Vibrio cholerae* reference pathway.


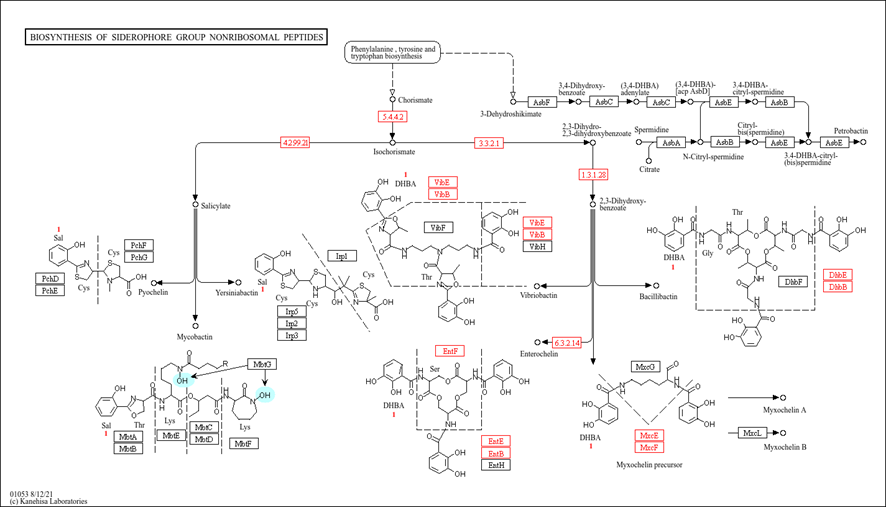


**Fig. S17.** Predicted siderophore biosynthesis pathways of *Chromobacterium violaceum* CNPSo 1954, annotated using The Kyoto Encyclopedia of Genes and Genomes (KEGG) database (http://www.kegg.jp).


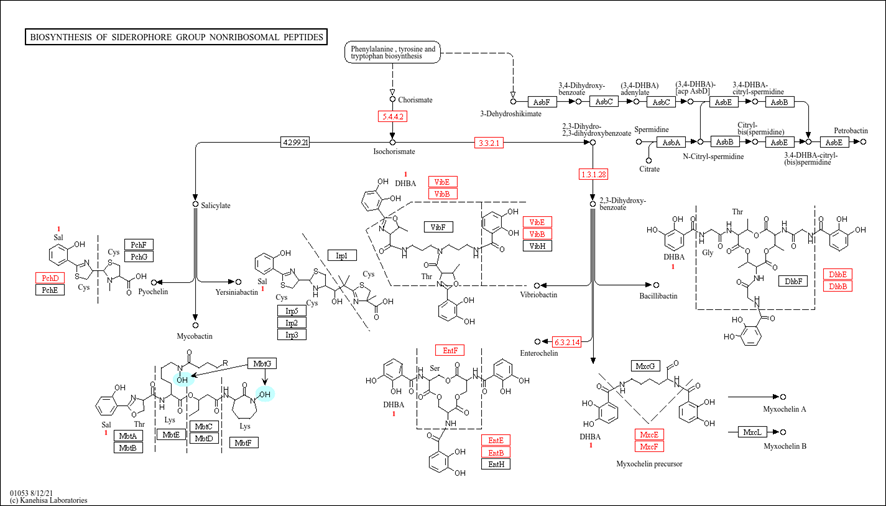


**Fig. S18.** Predicted siderophore biosynthesis pathways of *Pantoea agglomerans* CNPSo 2602, annotated using The Kyoto Encyclopedia of Genes and Genomes (KEGG) database (http://www.kegg.jp).


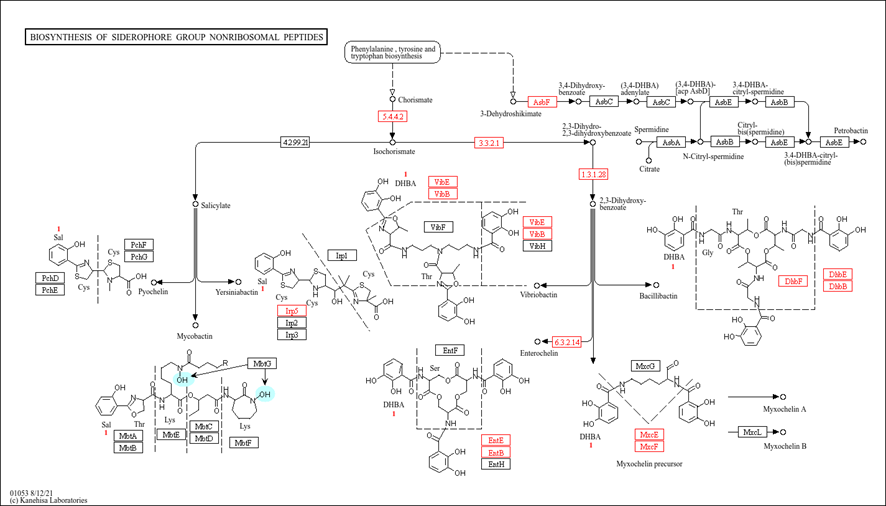


**Fig. S19.** Predicted siderophore biosynthesis pathways of *Bacillus velezensis* CNPSo 2657, annotated using The Kyoto Encyclopedia of Genes and Genomes (KEGG) database (http://www.kegg.jp).


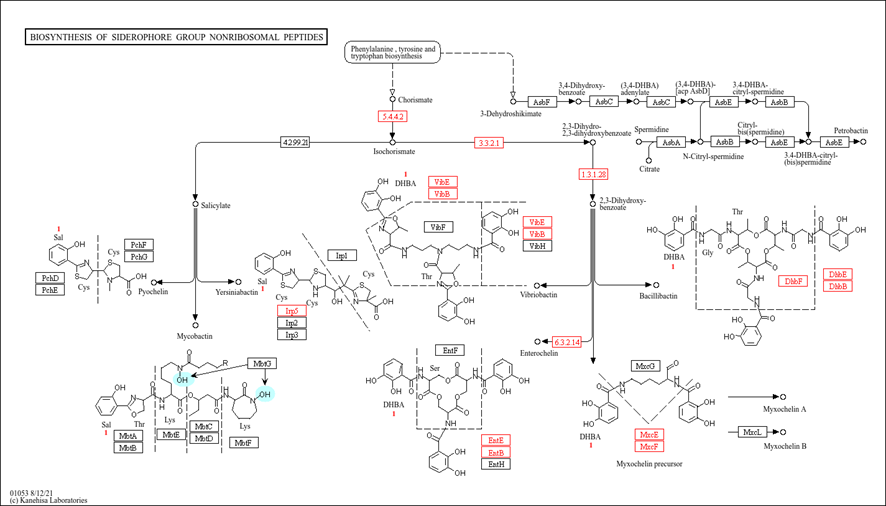


**Fig. S20.** Predicted siderophore biosynthesis pathways of *Bacillus safensis* CNPSo 2725, annotated using The Kyoto Encyclopedia of Genes and Genomes (KEGG) database (http://www.kegg.jp).


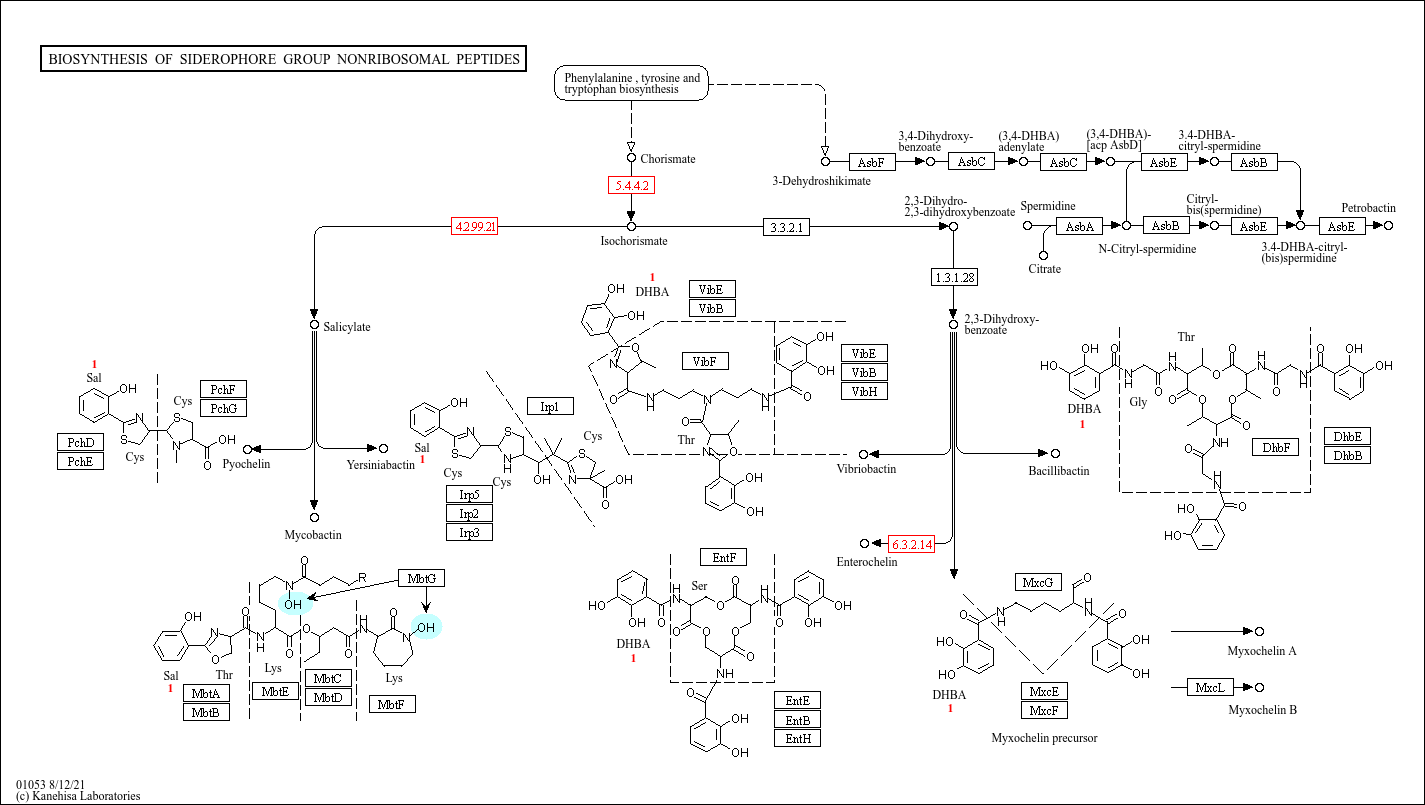


**Fig. S21.** Predicted siderophore biosynthesis pathways of *Pseudomonas* sp. CNPSo 2799, annotated using The Kyoto Encyclopedia of Genes and Genomes (KEGG) database (http://www.kegg.jp).


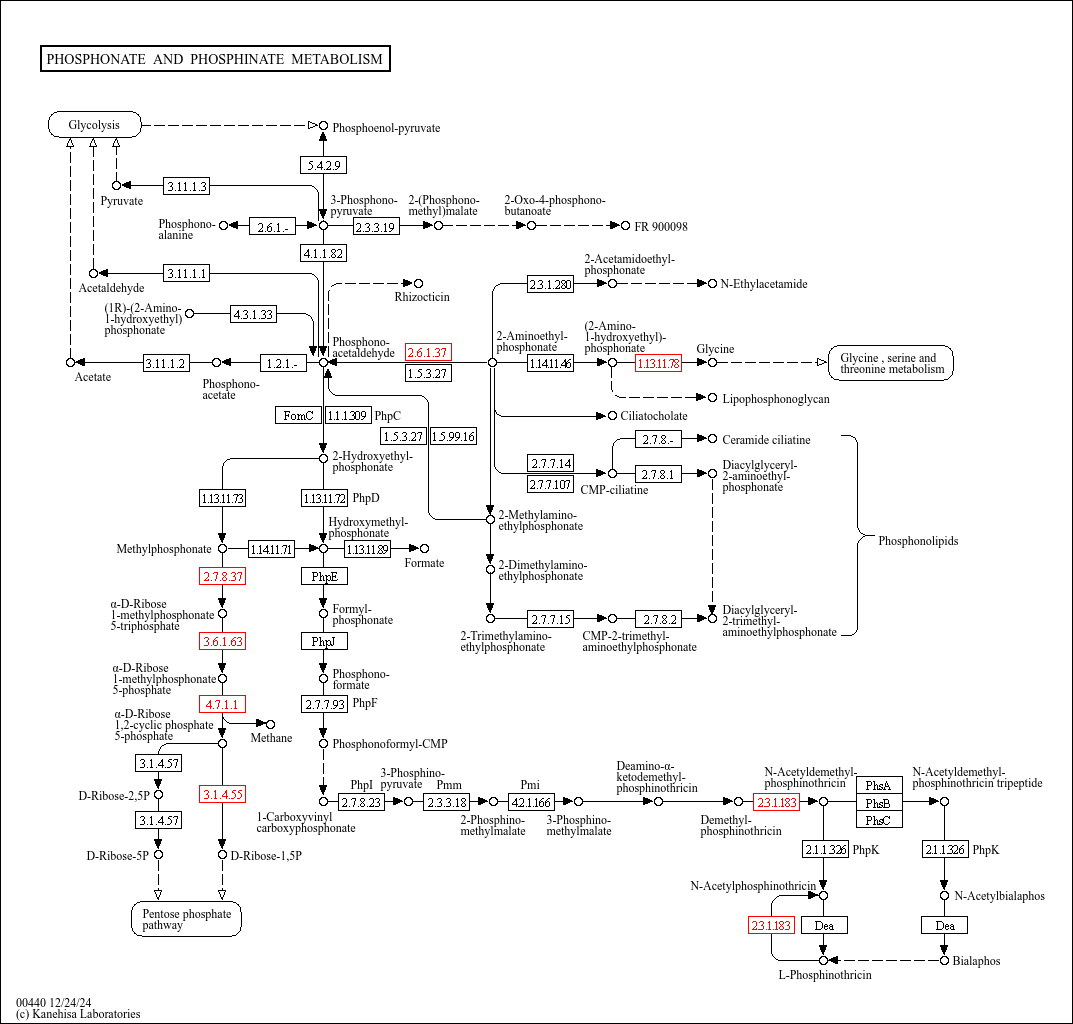


**Fig. S22.** Predicted phosphonate and phosphinate metabolism pathway, related with phosphorous solubilization of *Chromobacterium violaceum* CNPSo 1954, annotated using The Kyoto Encyclopedia of Genes and Genomes (KEGG) Database (http://www.kegg.jp).


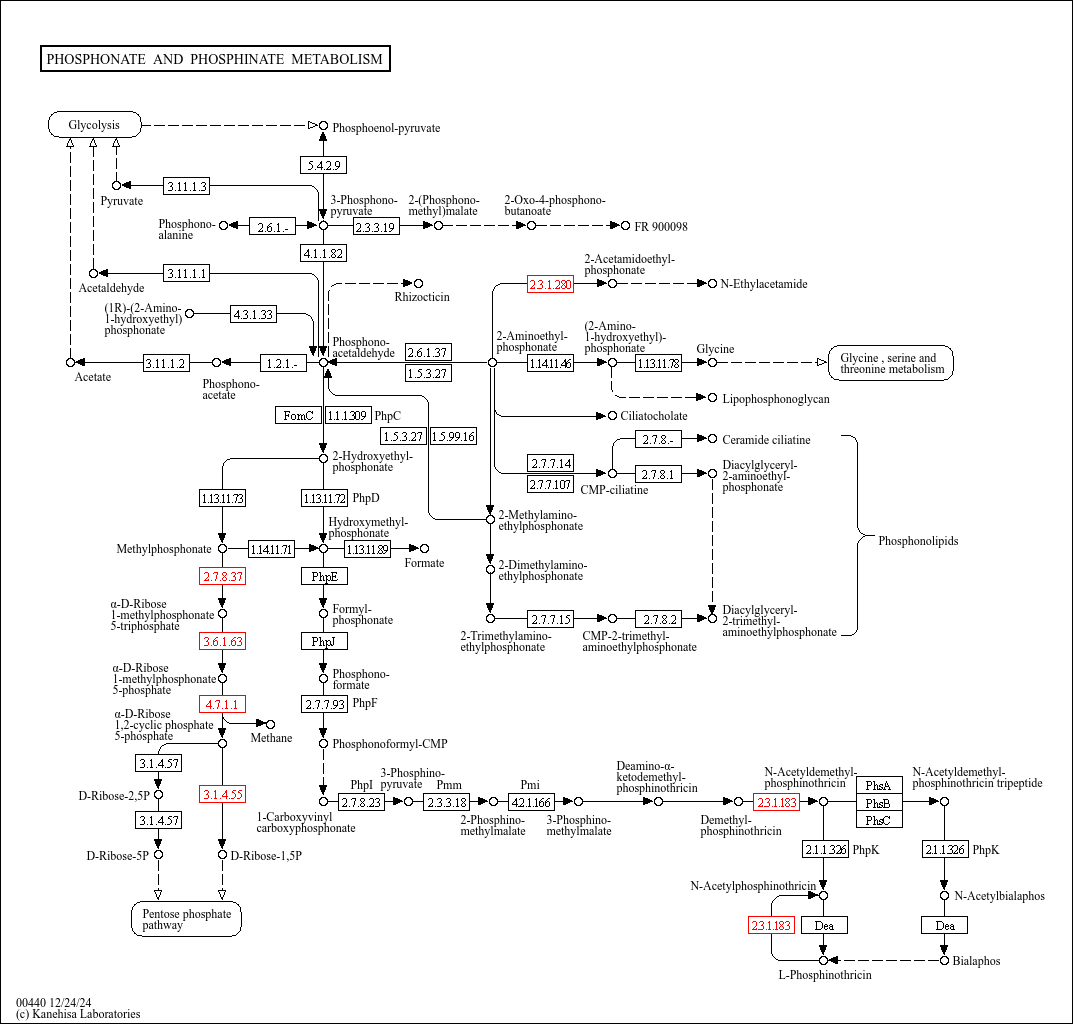


**Fig. S23.** Predicted phosphonate and phosphinate metabolism pathway, related with phosphorous solubilization of *Pantoea agglomerans* CNPSo 2602, annotated using The Kyoto Encyclopedia of Genes and Genomes (KEGG) Database (http://www.kegg.jp).


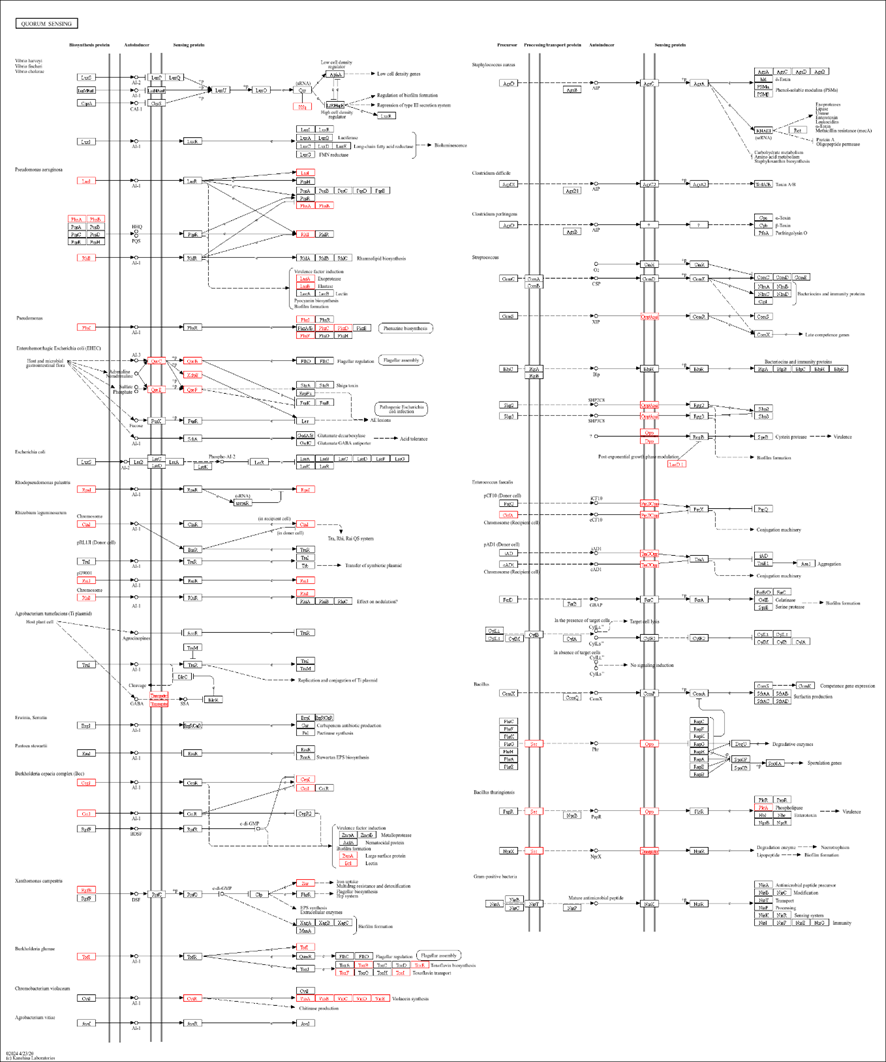


**Fig. S24.** Predicted quorum sensing (QS) pathway of *Chromobacterium violaceum* CNPSo 1954, annotated using The Kyoto Encyclopedia of Genes and Genomes (KEGG) Database (http://www.kegg.jp).


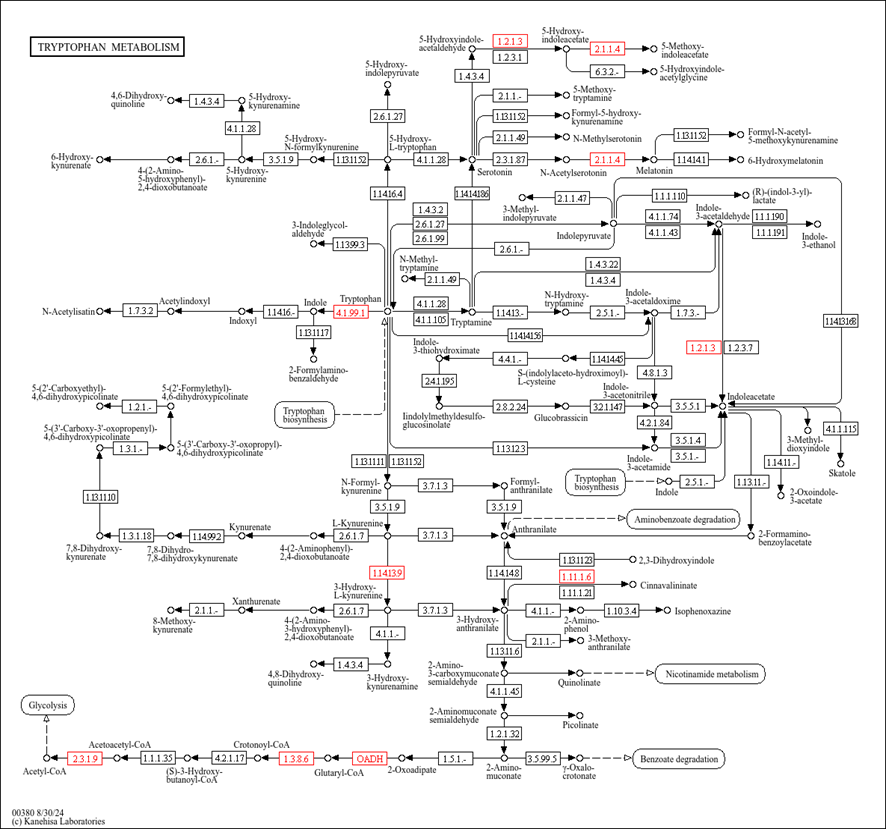


**Fig. S25.** Predicted tryptophan metabolism pathway of *Chromobacterium violaceum* CNPSo 1954, annotated using The Kyoto Encyclopedia of Genes and Genomes (KEGG) Database (http://www.kegg.jp).


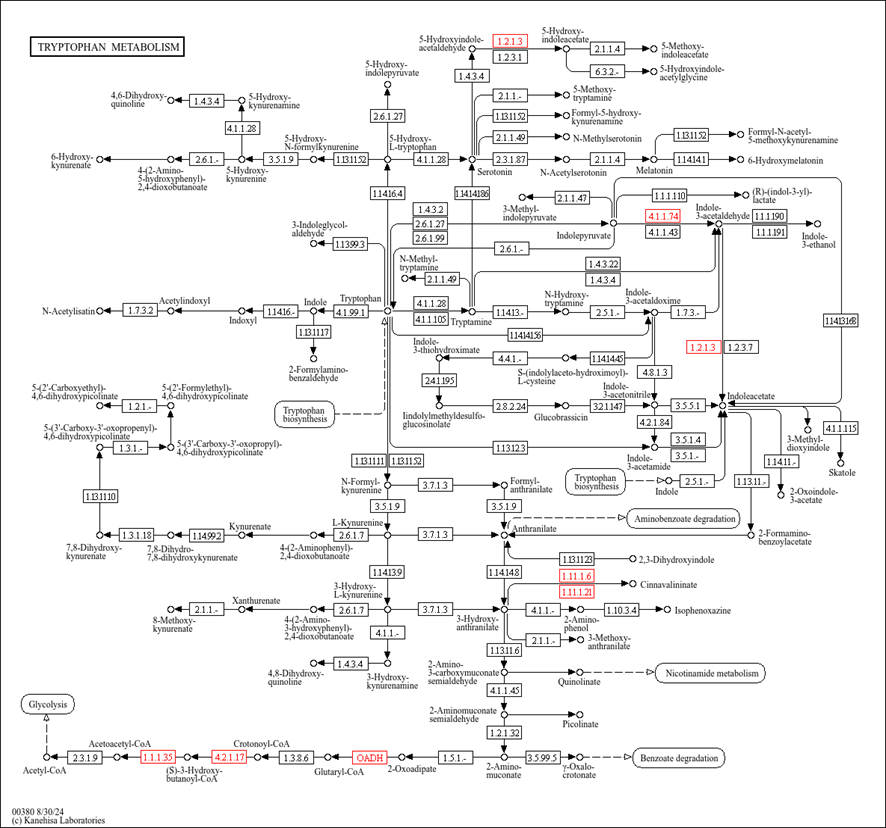


**Fig. S26.** Predicted tryptophan metabolism pathway of *Pantoea agglomerans* CNPSo 2602, annotated using The Kyoto Encyclopedia of Genes and Genomes (KEGG) Database (http://www.kegg.jp).


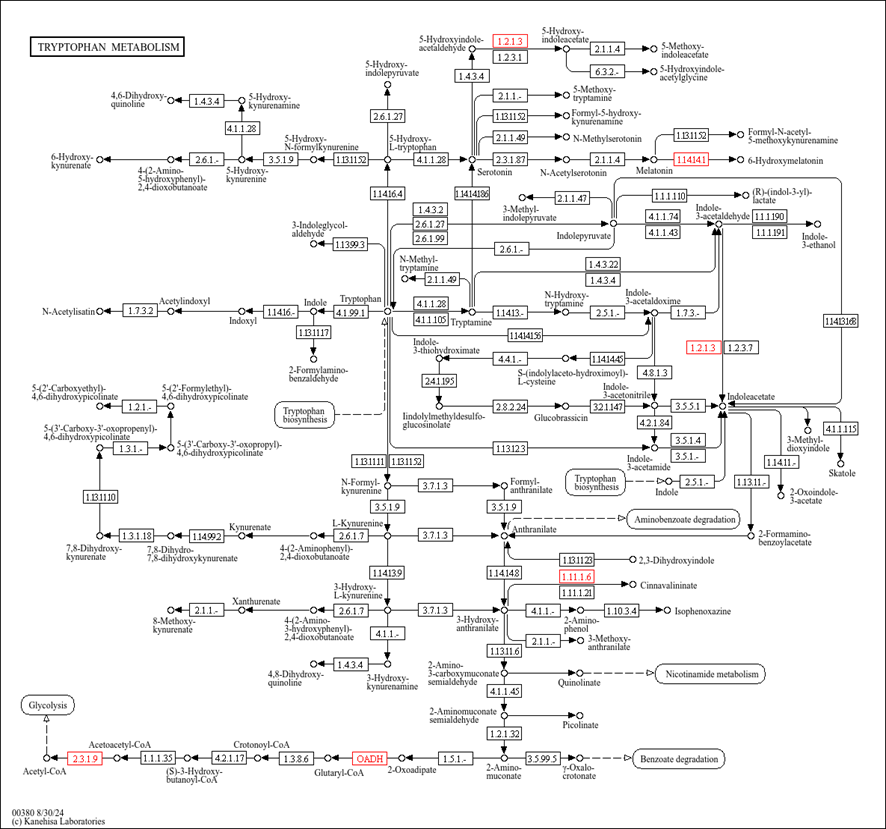


**Fig. S27.** Predicted tryptophan metabolism pathway of *Bacillus velezensis* CNPSo 2657, annotated using The Kyoto Encyclopedia of Genes and Genomes (KEGG) Database (http://www.kegg.jp).


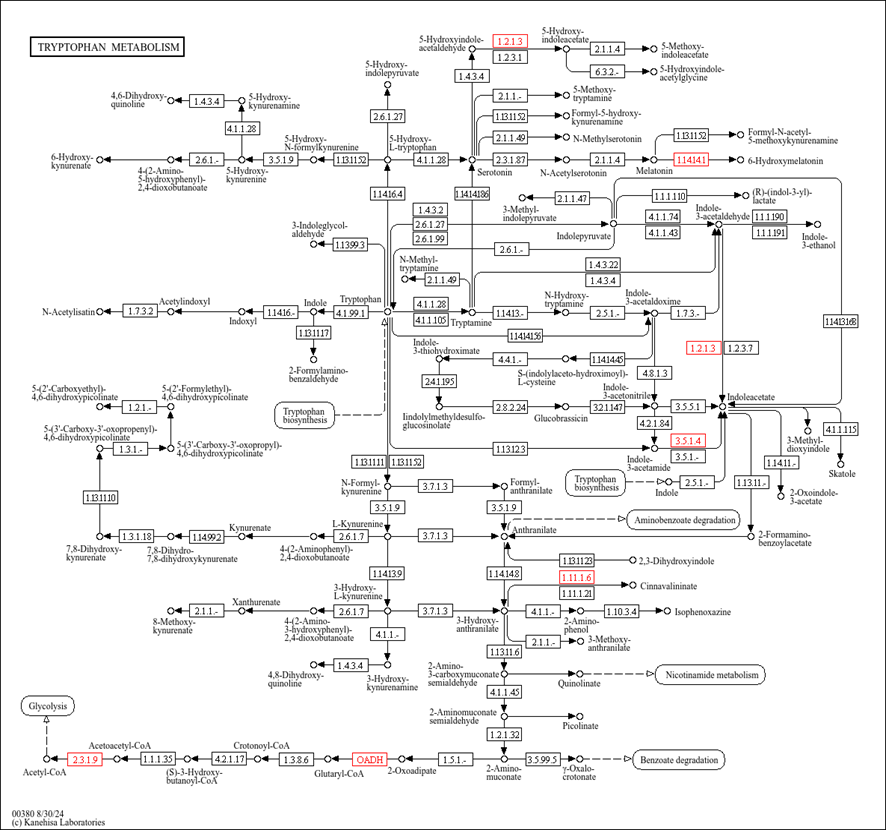


**Fig. S28.** Predicted tryptophan metabolism pathway of *Bacillus altitudinis* CNPSo 2658, annotated using The Kyoto Encyclopedia of Genes and Genomes (KEGG) Database (http://www.kegg.jp).


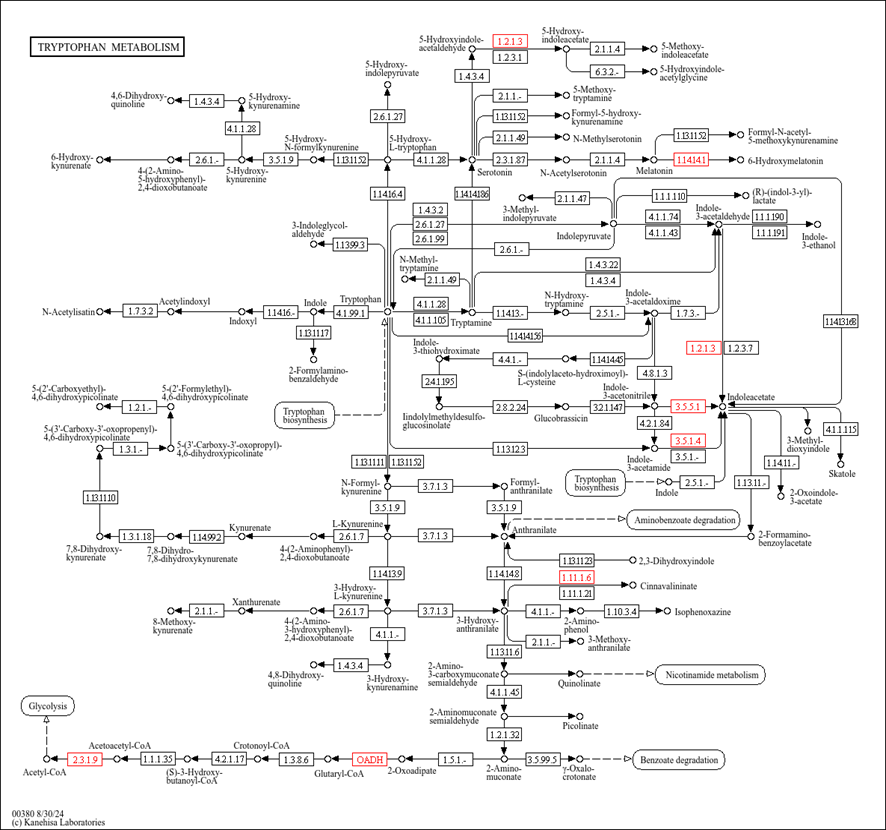


**Fig. S29.** Predicted tryptophan metabolism pathway of *Bacillus safensis* CNPSo 2725, annotated using The Kyoto Encyclopedia of Genes and Genomes (KEGG) Database (http://www.kegg.jp).


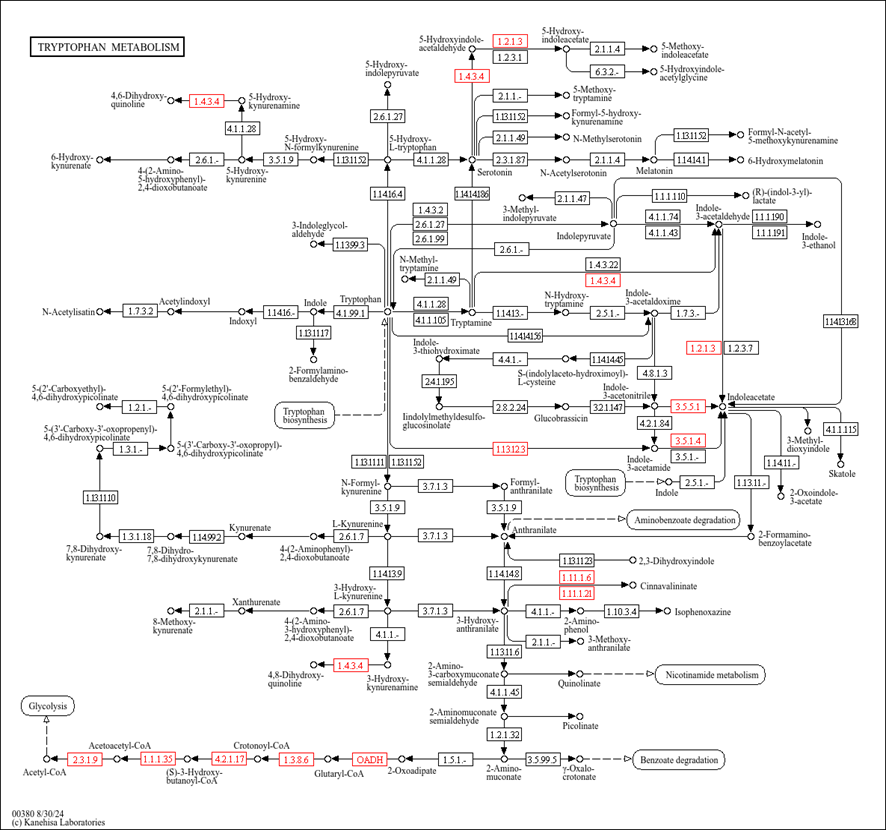


**Fig. S30.** Predicted tryptophan metabolism pathway of *Pseudomonas* sp. CNPSo 2799, annotated using The Kyoto Encyclopedia of Genes and Genomes (KEGG) Database (http://www.kegg.jp).


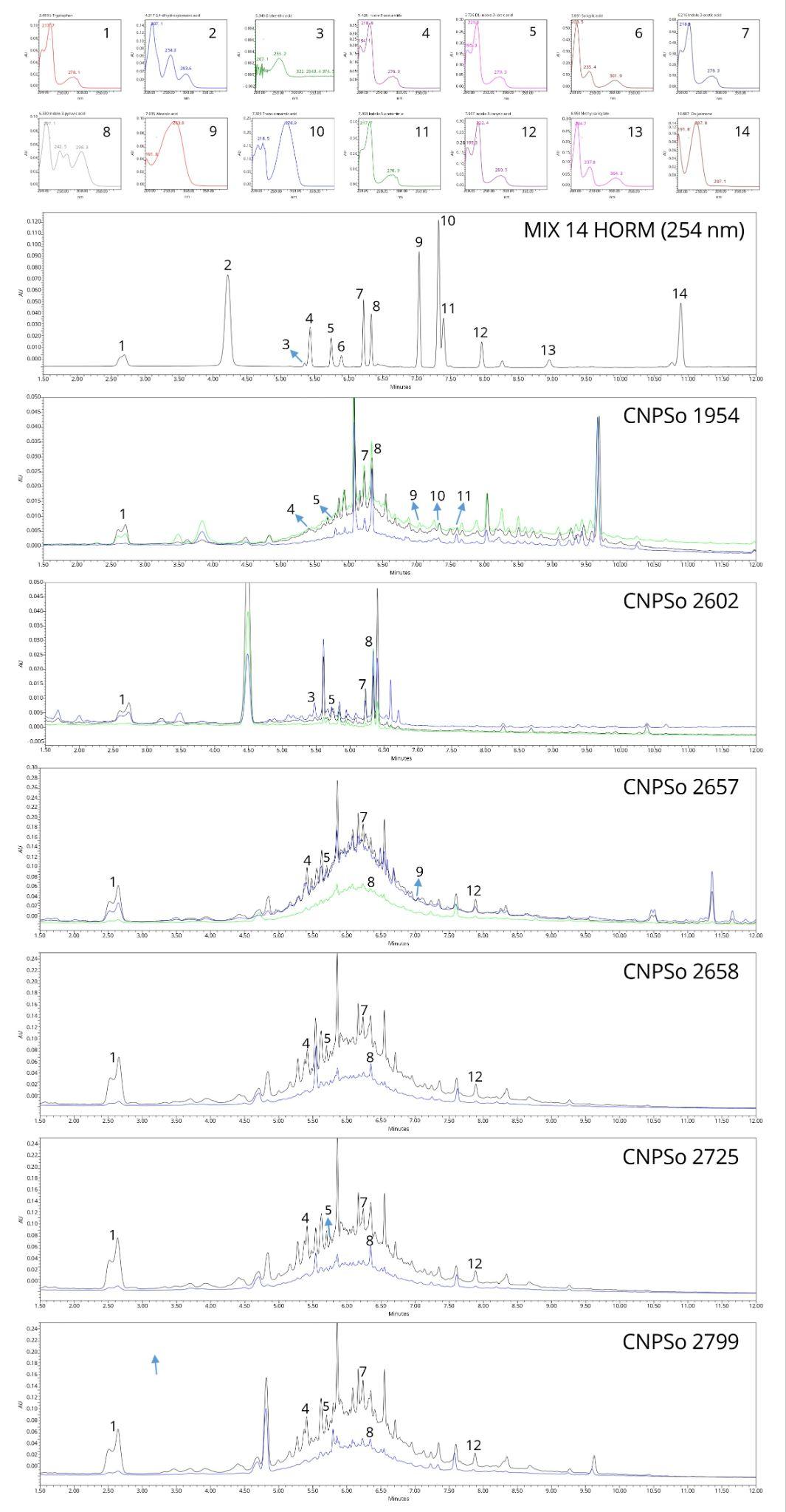


**Fig. S31.** Phytohormone detection analysis in cell-free metabolites (CFMs) of the plant growth-promoting bacterial (PGPB) strains CNPSo 1954, CNPSo 2602, CNPSo 2657, CNPSo 2658, CNPSo 2725, and CNPSo 2799. The compounds evaluated in triplicate were: (1) L-tryptophan; (2) 2,4-dihydroxybenzoic acid; (3) gibberellic acid*; (4) indole-3-acetamide; (5) DL-indole-3-lactic acid; (6) salicylic acid; (7) indole-3-acetic acid; (8) indole-3-pyruvic acid; (9) abscisic acid; (10) trans-cinnamic acid; (11) indole-3-acetonitrile; (12) indole-3-butyric acid; (13) methyl salicylate; and (14) cis-jasmone.

* Gibberellic acid (GA) did not reach the required sensitivity threshold and was therefore excluded from the analysis.
